# Identifying performance benchmarks and determinants for reproductive performance and calf survival using a longitudinal field study of cow-calf herds in western Canada

**DOI:** 10.1371/journal.pone.0219901

**Published:** 2019-07-18

**Authors:** Cheryl L. Waldner, Sarah Parker, John R. Campbell

**Affiliations:** Department of Large Animal and Clinical Sciences, University of Saskatchewan, Saskatoon, Saskatchewan, Canada; University of Illinois, UNITED STATES

## Abstract

The cow-calf industry in North America is in a period of rapid consolidation with corresponding increases in herd sizes and changes in management. The objectives of this study were to examine longitudinal data on reproductive performance in cow-calf herds and identify benchmarks for the most critical measures and important sources of differences among herds. To address these questions, a surveillance network was established in western Canada to collect data between 2013 to 2017 privately owned cow-calf herds during calving (n = 105 herds) and at pregnancy testing (n = 94 herds). Data were summarized for a number of indices of herd performance. However, the values considered to be most reliable and accurate were the percentage of females not pregnant when tested by a veterinarian, the percentage of calves dead within 24 hours of birth, and the percentage of calves dead from 24 hours to weaning. The mean and variation between herds for heifers, measured using standard deviation, was greater than for cows for: non-pregnancy (cows 6.8% (mean)±3.4%(SD), heifers 9.7%±8.2%), calf death from birth to 24 hours (cows 2.1%±1.6%, heifers 3.6%±4.5%), and calf death from 24 hours to weaning (cows 2.5%±2.4%, heifers 2.9%±3.9%). Benchmarks or performance targets derived from the 25th percentiles of these data for both cows and heifers were <5% for non-pregnancy risk and <1% for calf loss within 24 hours of birth. The suggested benchmark for calf loss from 24 hours to weaning was <2% for cows and <1% for heifers. All outcomes consistently displayed greater variation between herds as compared to year to year differences within herds with the exception of calf loss before 24 hours in cows. The timing of the start of breeding season was a consistent source of variation in risks of non-pregnancy and calf losses. Cows bred in April or earlier to start calving in late December or January were at increased risk of low pregnancy percentages (p<0.001) and calf losses at birth (p<0.04), as well as increased calf loss before weaning in both cows and heifers (p<0.02). There was also an increase in the risk of non-pregnancy for cows and heifers (p<0.001) where first exposure to breeding was not until July or August. In contrast, the risks of calf loss within 24 hrs of birth (p<0.001) and from 24 hrs to weaning in cows (p<0.02) first exposed to breeding in July and August were significantly lower than for herds that had earlier breeding seasons.

## Introduction

The Canadian beef industry is undergoing important changes characterized by decreasing herd numbers and increasing herd sizes [1]. The consolidation of smaller cow-calf herds into larger operations introduces biosecurity risks and the threat of diseases that can impact reproductive performance and animal health. As a further result of increasing herd sizes and the aging demographic of herd owners [2], there may also be changes to herd management to best utilize available labor and optimize input costs. One anecdotal example of these changes is the decision by many producers with larger herds to delay breeding to allow for later calving on pastures and, therefore reduce the need for labor to manage newborns in extreme cold and winter storms during the calving season.

To assess the potential impacts from ongoing industry changes to reproductive performance, surveillance data are needed. These data must be collected on an appropriate geographic scale to consider regional variation in climate, nutrition and management decisions on herd performance. While other one time surveys have been successful in providing data to inform performance expectations [3–9], there is limited understanding of how herd productivity fluctuates from year-to-year in response to changing weather patterns, feed quality, and management decisions. In addition to examining trends over time, regional surveillance data can also be used to develop benchmarks against which producers and veterinarians can evaluate individual herd performance and set goals for improvement.

More than 80% of Canadian cow-calf production is located in the western provinces of Alberta, Saskatchewan and Manitoba [10]. In response to industry needs, a longitudinal surveillance network was established in western Canada and began data collection in the first quarter of 2014. Each year participants were asked to complete three to four surveys which included requests for basic information on herd inventory, breeding management and reproductive performance. The initial objectives of this analysis were to summarize the year to year variation in reproductive performance collected from 2013 to 2017, to identify benchmarks for the most useful measures, and to compare these values to the results of previous studies. The secondary objectives were to identify important risk factors for differences in reproductive outcomes among network participants and study years.

## Materials and methods

### Herd recruitment and data collection

Enrollment targets for cow-calf herds within Alberta, Saskatchewan, and Manitoba were established using data from 2011 Census of Agriculture [11]. These were determined from the proportion of cow-calf herds located in each province as well as the targeted geographic distribution of moderate (100 to 300 cow-calf pairs) and larger sized herds (>300 cow-calf pairs).

Researchers identified veterinary practices with beef cow-calf herd clients in each region. Veterinarians then contacted clients who pregnancy tested and reported keeping at least basic calving and production records. Interested producers were mailed a consent form and survey to collect baseline data from calving season 2013. This study was approved by the University of Saskatchewan Animal Research Ethics Board (#20140003) and the University of Saskatchewan Behavioural Research Ethics Board (#14–07).

### Survey design

At the start of the program in 2014 all of the questions for one production cycle were consolidated on a single questionnaire to obtain historical data from breeding in 2013. Data collection for herd productivity in each subsequent year to 2017 was split to focus on calving and breeding, followed by autumn pregnancy testing and weaning periods. The questions provided to producers were summarized and provided in S1 File.

Questions at each production period from 2013 to 2017 asked about the number of events that occurred, and also about the numbers of animals that could have experienced that event. Questions were also included that asked about additions and losses to the herd since the last production period. Participants were asked to identify if they had separate records for heifers and to provide numbers for cows and heifers separately if possible.

Production questions asked about numbers of cows that aborted (observed abortions and cows diagnosed pregnant that failed to calve), total number of cows calving, number of twins, number of calves dead within the first 24 hours, and number of calves that died from 24 hours to weaning. Producers were also asked for the number of cows and heifers exposed to breeding (natural and AI), and number of animals not pregnant and the number that were checked by a veterinarian to test for mid-gestation pregnancy status. Producers were also asked to indicate if they pregnancy tested their whole herd or just a portion and, if just a portion, the reason why.

A first reminder for each questionnaire was sent out between 2 to 4 weeks after each questionnaire for any participants who had not yet responded. Reminders were sent on an ongoing basis to participants, along with the next delivered communication. For all questionnaires, a minimum of 3 reminders was sent to participants that had not yet responded. Participants were considered to have dropped from the study, either after they communicated they wished to withdraw, or if no responses were received for approximately 1 production cycle.

Between February 2014 and November 2016, 122 producers in total were recruited, provided consent forms and contributed at least one survey, 22 stopped responding to requests to return surveys at various points from autumn of 2014 through the autumn of 2017. Where possible producers who withdrew were replaced. Of the original 105 producers recruited in 2014, 3 withdrew in year 1 (3%), 9 withdrew in year 2 (10%), 4 withdrew in year 3, (4%), and 4 withdrew in year 4 (5%). Five producers were recruited between March and July 2017 and 1 withdrew in 2017. Twelve more producers were recruited in 2016 and 2 subsequently withdrew.

### Data entry and statistical analysis

Survey responses were recorded on a spreadsheet and checked for accuracy. Outcome variables derived from survey responses included: the percentage of females not pregnant at pregnancy testing, the percentage of females retained until calving that aborted, the percentage of calves born full term that died within the 24 hours of birth, and the percentage of calves alive at 24 hours that died between 24 hours of birth and weaning. The number of observations available for analysis were reported with descriptive summaries of herd-level data for each metric. Study data are available in S2 File and S3 File.

Factors considered and included in all statistical analysis were location by province, whether any purebred cattle were sold (self-identified seed stock producer), herd size at either breeding or calving as appropriate to the outcome, month when the breeding season started, and year of data collection. The start of breeding season was chosen as a management factor as it is easier for producers to define when compared to the start of calving season. The start of breeding season is a more easily manipulated management decision than the start of calving season which may vary depending on gestation lengths, the birth of premature calves or calves from purchased cows. A conversion for month when breeding season began to the start of calving season is provided in Table 1.

**Table 1:** Expected calving dates for different start times for the breeding season assuming a 282 gestation period.

Start of breeding season	Start of calving season
March 15^th^	December 22^nd^
April 15^th^	January 22^nd^
May 15^th^	February 21^st^
June 15^th^	March 24^th^
July 15^th^	April 23^rd^
August 15^th^	May 24^th^

As estimates for all listed factors were of interest in this analysis and also considered as potential confounders of other estimates, the complete set of variables was retained in the multivariable analyses for each outcome. Each outcome was examined separately for data from heifers and from cows. Generalized estimating equations with a logit link function and binomial distribution were used to account for repeated measures among herds and provide population-averaged estimates of effect for potential risk factors. The counts of the outcome of interest for each of the herds for each year were the numerators for the multivariable analyses. The total numbers of animals at risk of each outcome for each of the herds for each year were the denominators. Results were reported as odds ratios (OR) with 95% confidence limits (95%CI). Results for heifers and cows were analyzed and reported separately; no other effect modifiers were examined as this report was focussed on the description of benchmarking data. *P*< 0.05 was considered statistically significant.

Three level subject-specific random-intercept logistic regression models were then used to estimate the proportion of total variation for each outcome that was explained by the differences among herds as compared to the proportion of variation explained by the differences across years within individual herds during the study period. Models for nonpregnancy, abortion, death within 24 hours of birth, and death from 24 hours of birth to weaning for cows and then for heifers included a fixed effect for study year and random intercepts for herd identification and the interaction of herd identification and study year. Variance components were estimated using a latent variables approach with the following formula [12]:

$$VC=\frac{\sigma^{2}}{\left(\sigma^{2}+\pi^{2}/3\right)}$$ (1)

## Results

### Profile of herds providing whole-herd pregnancy testing data

The pregnancy data presented here are limited to herds that reported testing all cows and/or all heifers for at least one year during the study (Tables 2 and 3). Fifty-six other herd observations included pregnancy test data for only a portion of mature cows and were not included in subsequent analysis of pregnancy test data. Similarly, an additional 61 herd reports did not provide complete data for all heifers and were not included.

**Table 2:** Description of herds with available pregnancy testing data where herd owner reported whole herd testing from 2014 to 2017.

Herd attributes for pregnancy testing data	Cows	Heifers
Number females pregnancy tested per herd		
• Median	220	47
• 5^th^ to 95^th^ percentile	91 to 851	12 to 201
Total number of female observations^1^	82,186	18,508
Number of herd observations^1^	276	270
Number of herds with at least 1 herd record^1^	94	93
% of herds with at least one AI record	21% (20/94)	22% (20/93)
• Commercial only	10% (7/72)	14% (10/71)
• At least some purebred sales	59% (13/22)	45% (10/22)
% of all annual herd observations with at least some AI	16% (42/268)	17% (44/263)
• Commercial only	6.3% (13/205)	10% (20/200)
• At least some purebred sales	46% (29/63)	38% (24/63)
% females bred at least once by AI	3.6% (2891/80,241)	12% (2241/18,443)
• Commercial only	2.0% (1186/59,512)	8.1% (1173/14,482)
• At least some purebred sales	8.2% (1705/20,729)	27% (1068/3961)

^1^ Limited to years where all cows / all heifers were reported pregnancy tested.

**Table 3:** Production indices for herds from 2014–2017.

Herd Summary Statistics	% females not pregnant when tested		Abortion Cumulative Incidence		Cumulative incidence of calf death from birth– 24 hours		Cumulative incidence of calf death from 24 hours to weaning	
	Cows	Heifers	Cows	Heifers	Cows	Heifers	Cows	Heifers
Mean (SD)	2.1%	3.6%	0.8%	1.3%	2.1%	3.6%	2.5%	2.9%
Standard deviation	1.6%	4.5%	0.9%	2.3%	1.6%	4.5%	2.4%	3.9%
5^th^ percentile	0.0%	0.0%	0.0%	0.0%	0.0%	0.0%	0.0%	0.0%
25^th^ percentile	1.0%	0.0%	0.0%	0.0%	1.0%	0.0%	1.1%	0.0%
Median	6.2%	8.3%	0.7%	0.0%	1.8%	2.6%	1.9%	1.5%
75^th^ percentile	8.9%	12.9%	1.2%	2.1%	2.8%	5.3%	3.3%	4.6%
95^th^ percentile	12.8%	24.1%	2.6%	5.6%	4.8%	11.5%	6.9%	10.4%
Observations	276	270	353	338	359	345	350	332
Number of herds	94	90	105	104	105	105	105	105

The total number of herds providing complete pregnancy testing data for cows from at least one year during 2014 to 2017 was 94 (Table 2). Of these, 33 (35%) were from Saskatchewan, 44 (47%) from Alberta (including 1 north east BC herd), and 17 (18%) were from Manitoba. The herds were described as either commercial operations (77%, 72/94) or selling at least some purebred cattle (23%, 22/94). Only 4 herds (4%) were described as being exclusively purebred.

Corresponding information on the start of breeding season was available for 94 of these herds for 2014 to 2017. The earliest dates for the start of breeding season for the cows reported from 2014 to 2017 from these herds were April or earlier (27%), May (16%), June (26%), July or August (32%). The latest dates reported during this period for the start of breeding season for the cows from these herds were April or earlier (16%), May (19%), June (24%), July or August (39%).

The earliest breeding dates reported for the start of breeding season for the heifers from these 93 herds from 2014 to 2017 were in April or earlier (27%), May (19%), June (29%), July or August (25%). The latest dates for the start of breeding season reported for the heifers from these herds during this period were in April or earlier (18%), May (17%), June (32%), July or August (32%).

Herds that bred ≥ 300 cows were more likely than smaller herds to start breeding season in July or August as compared to earlier (odds ratio (OR) 2.1, 95%CI 1.2–3.5, *P =* 0.005), after accounting for differences between commercial herds and herds with some purebred sales (*P =* 0.42), province (*P =* 0.61), and year of data collection (*P =* 0.98).

Herds that bred < 300 cows were more likely than larger herds to start breeding season in April or earlier as compared to later [OR 2.2, 95%CI 1.0–4.5, *P =* 0.04] as were herds with some purebred sales (OR 2.8, 95%CI 1.1–7.2, *P =* 0.03) after accounting for province (*P =* 0.06) and year of data collection (*P =* 0.06).

### Pregnancy testing 2014–2017

The mean percentage of cows that were not pregnant when tested were slightly lower than the percentage of heifers that were not pregnant (Table 3). The percentage of heifers that were not pregnant also varied more widely than for cows (Table 3). Across all herd observations, the 25^th^ percentile for the risk of non-pregnancy when tested was 4.3% for cows and 4.2% for heifers. That is, 25% of herds pregnancy tested achieved less than 4.3% non-pregnant cows and 4.2% heifers, respectively.

The percentage of total variation in non-pregnancy risk of cows attributed to differences between herds was 3.6% and the percentage of total variation attributed to year to year differences within herds for cows was calculated to be 2.7%. The percentage of total variation in non-pregnancy risk of heifers attributed to differences between herds was 7.9% and the percentage of total variation attributed to year to year differences within herds for heifers was calculated to be 5.8%.

### Factors associated with risk of non-pregnancy in cows

The model examining risk factors for non-pregnancy in cows included 276 herd observations with complete cow herd outcome and risk factor data from 94 different herds.

Cows first exposed to breeding in April or earlier were less likely to be pregnant (OR 1.2 95%CI 1.1–1.3, p<0.001) than cows from herds where the breeding season started between May and June. In addition, cows from herds that started the breeding season in July or August were also less likely to be pregnant (OR 1.3 95%CI 1.2–1.4 p<0.001) than cows from herds that started the breeding season in May or June. There was no difference in the risk of non-pregnancy between cows bred in April or earlier and July and August (*P =* 0.26) and between cows bred in May and June (*P =* 0.31).

Cows from herds in Manitoba were less likely to be pregnant compared to herds from Saskatchewan (OR 1.3 95%CI 1.1–1.4 p<0.001) and Alberta (OR 1.3 95%CI 1.2–1.4 p<0.001).

Cows were less likely to be pregnant in 2017 than in 2015 (*P =* 0.02) (Table 4).

**Table 4:** Mean production indices (standard deviation) of herds by year of testing.

		2014	2015	2016	2017
% not pregnant at pregnancy testing	Cows	6.7% (2.7) N = 69	6.6%(3.9) N = 75	6.8%(3.5) N = 73	7.1%(3.7) N = 59
	Heifers	9.1%(7.3) N = 65	9.6% (8.5) N = 75	10.1% (7.9) N = 72	9.9% (9.1) N = 58
Abortion Cumulative Incidence	Cows	1.0% (1.0) N = 83	0.7% (0.7) N = 93	0.8% (0.9) N = 91	0.9% (0.8) N = 86
	Heifers	1.6% (2.5) N = 79	0.9% (1.9) N = 90	1.5% (2.9) N = 86	1.4% (2.0) N = 83
Cumulative incidence of calf death from birth– 24 hrs	Cows	2.4% (1.6) N = 84	2.0% (1.4) N = 93	1.9% (1.4) N = 95	2.1% (1.9) N = 87
	Heifers	4.7% (5.7) N = 79	3.8% (4.3) N = 90	3.0% (4.0) N = 92	2.9% (3.8) N = 84
Cumulative incidence of calf death (24 hrs-weaning)	Cows	2.7% (2.8) N = 84	2.4% (2.0) N = 94	2.3% (1.8) N = 93	2.9% (2.9) N = 90
	Heifers	3.4% (4.7) N = 78	2.1% (2.8) N = 90	3.2% (4.1) N = 90	2.8% (3.8) N = 90

Cows from commercial herds (OR 1.2 95%CI 1.1–1.3 *P =* 0.002) were less likely to be pregnant than cows from herds that reported selling at least some purebred cattle. The total number of females pregnancy tested was not associated with the risk of non-pregnancy in cows (*P =* 0.10).

### Factors associated with risk of non-pregnancy in heifers

The model examining risk factors for non-pregnancy in heifers included 265 observations with complete heifer herd data from 93 herds.

Similar to what was observed with the cows, heifers from herds that started the breeding season in July or August were less likely to be pregnant (OR 1.4 95%CI 1.2 to 1.5 p<0.001) than heifers from herds that started breeding season in June or earlier. However, in contrast to what was seen with the cows, herds that started breeding their heifers early were not at greater risk. Heifers first exposed to breeding in April or earlier were more likely to be pregnant (OR 1.4 95%CI 1.2 to 1.8, p<0.001) than heifers from herds where the breeding season started between May and August. There was no difference in the risk of non-pregnancy between heifers bred in May and June (*P =* 0.25).

Heifers from herds in Manitoba were less likely to be pregnant compared to herds from Saskatchewan (OR 1.4 95%CI 1.2–1.7 p<0.001) and Alberta (OR 1.4 95%CI 1.2–1.7 p<0.001).

The percent of heifers that were not pregnant did not differ significantly by year (*P =* 0.27) (Table 4).

There was no difference between heifers from commercial herds (*P =* 0.84) compared to those that reported selling at least some purebred cattle. Heifers were, however, less likely to be pregnant from larger herds that pregnancy tested >300 females (OR 1.2 95%CI 1.0–1.4 *P =* 0.016).

### Profile of herds providing breeding or calving season data for at least one year during the study

The total number of herds providing data for at least one year during 2013 to 2016 including herd size at breeding was 111 (Table 5). Of these, 36 (32%) were from Saskatchewan, 56 (51%) from Alberta (including 1 north east BC herd), and 19 (17%) were from Manitoba. The herds were described as either commercial operations (75%, 83/111) or selling at least some purebred cattle (25%, 28/111). Only 6 herds (5%) were described as being exclusively purebred.

**Table 5:** Summary of available herd data for cows and heifers for breeding and calving seasons for 2013 to 2016.

	Cows	Heifers
Earliest month start of breeding	February	February
Latest month start of breeding	August	August
Herd size at breeding		
• Median	220	45
• 5^th^ to 95^th^ percentile	97 to 817	12 to 218
Total number of female observations	110,327	24,259
Number of herd observations	361	348
Number of herds with at least 1 record	111	109
• Commercial only	83	82
• At least some purebred sales	28	27
**Herd attributes for calving season**		
Earliest month with first calf born	December	December
Latest month with first calf born	May	May
Earliest month with last calf born	January	March
Latest month with last calf born	July	August
Length of calving season		
• Median (days)	85	63
• 5th to 95th percentile	56 to 150	34 to 121
Herd size (at calving)		
• Median	187	36
• 5th to 95th percentile	87 to 705	12 to 173
Total number of female observations	94,505	18,430
Number of herd observations	357	342
Number of herds with at least 1 record	105	105

Corresponding information on the start of breeding season was available for 110 of these herds for 2013 to 2016 (Table 5). The earliest dates reported for the start of breeding season for cows from these herds from 2013 to 2016 were April or earlier (25%), May (17%), June (28%), July or August (29%). The latest reported dates for the start of breeding season for cows during this period were April or earlier (17%), May (19%), June (26%), July or August (37%).

The earliest breeding dates reported for heifers from these 109 herds from 2013 to 2016 were in April or earlier (25%), May (22%), June (31%), July or August (22%) (Table 5). The latest reported dates for the start of the breeding season for heifers from these herds during this period were in April or earlier (18%), May (18%), June (30%), July or August (34%).

The average percentage of heifers across all annual observations for herds providing breeding data was 22.6% (SD 10.7%); the median was 20.3% (5^th^ percentile 8.0%, 95^th^ percentile 42.2%).

The total number of cows and heifers exposed to breeding and present at calving varied from year to year (Table 6).

**Table 6:** Number of cows and heifers exposed to breeding and reported at calving (2014 to 2017).

		2013–2014	2014–2015	2015–2016	2016–2017
Number exposed to breeding	Cows	93	89	93	86
	Heifers	86	86	91	85
Number present at start of calving season	Cows	83	94	94	86
	Heifers	78	90	91	83

### Abortion risk for cows and heifers 2014–2017

Abortion risk or cumulative incidence was calculated as the total number of reported abortions as a percentage of the total number of cows and heifers retained in the herd till calving (Tables 3 and 4). Abortions were defined as loss between end of breeding season and calving where the fetus was not considered to be full term either observed by the producer or determined based on cows reported as pregnant that did not calve. The mean percentage of cows that were reported to have aborted was slightly lower than the percentage of heifers (Table 3). The percentage heifers that aborted also varied more than for cows (Table 3). The percentage of cows and heifers reported to have aborted was 0% for one-quarter of herd observations.

The model for abortion accounted for cows contained 325 observations with complete data from 102 herds and the model for abortion in heifers included 311 observations with complete data from 101 herds.

There was no association between the start of breeding season and the risk of abortion for either cows (*P =* 0.07) or heifers (*P =* 0.86)

The risk of abortion from Alberta herds was higher than from Saskatchewan for cows (*P =* 0.02), but there was no difference across provinces for heifers (*P =* 0.27).

The risk of abortion was higher in 2014 than in 2015 for cows (*P =* 0.01) and heifers (*P =* 0.03) and it was also higher in 2017 than in 2015 (p<0.001) for cows and for heifers (*P =* 0.01) (Table 4). Finally, the risk of abortion was higher in 2016 than in 2015 (*P =* 0.01), but just for heifers.

There was no association between the risk of abortion based on whether herd size was >300 cows for cows (*P =* 0.34) or heifers (*P =* 0.70).

The risk of abortion was also higher for cows (OR 1.4 95%CI 1.1–1.6 p<0.001) and for heifers (OR 1.5 95%CI 1.1–2.1 *P =* 0.01) from herds that sold at least some purebred cattle than those that did not.

The percentage of total variation in abortion risk of cows attributed to differences between herds was 4.3% and the percentage of total variation attributed to year to year differences within herds for cows was calculated to be 4.9%. The percentage of total variation in abortion risk of heifers attributed to differences between herds was 5.3% and the percentage of total variation attributed to year to year differences within herds for heifers was calculated to be 0.1%.

### Risk of death from birth to 24 hours for cows and heifers 2014–2017

Risk of death from birth to 24 hours was calculated as the total number of calves dead at or within 24 hours of birth as a percentage of the total number of calves born that were considered to be full term (not abortions) (Tables 3 and 4). The mean percentage of calves dead from birth to 24 hours was slightly lower for cows than for heifers (Table 3). The percentage calves reported dead from birth to 24 hours for heifers also varied more widely than for cows (Table 3). The percentage of calves that were reported dead from birth to 24 hours from cows was <1.0% for one-quarter of herd observations and was 0% for heifers.

The models for calves dead from birth to 24 hours for cows included 330 observations with complete data from 102 herds and the model for heifers included 318 observations with complete data from 102 herds.

Risk of calf death from birth to 24 hours was lower for cows first exposed to breeding in July or August than those bred in May or earlier (OR 1.4 95%CI 1.2–1.6 p<0.001). There was no difference in risk of calf death from birth to 24 hours for cows bred in June and those bred in July and August (*P =* 0.13) or between those bred in May or June (*P =* 0.41). The risk of calf death from birth to 24 hours was also higher for cows bred in April or earlier than for cows bred in May (OR 1.2 95%CI 1.0–1.5 *P =* 0.04) or June (OR 1.3 95%CI 1.1–1.6 *P =* 0.01). There was no association between the month that breeding began and the risk of calf death from birth to 24 hours in heifers (*P =* 0.66).

There was also no significant difference in the risk of calf death from birth to 24 hours among provinces for calves from cows (*P =* 0.87) or heifers (*P =* 0.51).

Risk of calf death from birth to 24 hours was significantly lower in 2016 than in 2014 (*P =* 0.001) or 2015 (*P =* 0.03) for cows (Table 4). Risk of calf death from birth to 24 hours was significantly higher in 2014 than in 2016 (*P =* 0.03) or 2017 (*P =* 0.02) for heifers.

There was no significant difference in risk of calf death from birth to 24 hours based on whether the herd size at calving was >300 for cows (*P =* 0.12) or for heifers (*P =* 0.07) and whether or not the herd sold any purebred cattle for cows (*P =* 0.95) or for heifers (*P =* 0.61).

The percentage of total variation in risk of calf death from birth to 24 hours for cows attributed to differences between herds was 4.3% and the percentage of total variation attributed to year to year differences within herds for cows was calculated to be 4.5%. The percentage of total variation in risk of calf death from birth to 24 hours for heifers attributed to differences between herds was 7.2% and the percentage of total variation attributed to year to year differences within herds for heifers was calculated to be 5.1%.

### Risk of calf loss from birth to weaning for cows and heifers 2014–2017

Risk of calf loss from birth to weaning was calculated as the total number of calves dead from 24 hours of birth to weaning as a percentage of the total number of calves alive at 24 hours (Tables 3 and 4). The mean percentage of calves that died between 24 hours of birth and weaning was similar for cows than for heifers (Table 3). However, the percentage calves reported as dead before weaning for heifers varied more than for cows (Table 3). The percentage of calves died from cows was < 1.1% for one-quarter of herd observations and was 0% for heifers.

The models for calf death loss between 24 hours and weaning for cows included 324 observations with complete data from 102 herds and the model for heifers included 308 observations with complete data from 101 herds.

Calf death loss between 24 hours and weaning was highest for cows exposed to breeding in April or earlier with the risk lowering significantly for cows first exposed to breeding in May (OR 1.2 95%CI 1.0–1.5 *P =* 0.02). The risk significantly lowered again from May to June (OR 1.2 95%CI 1.0–1.5 *P =* 0.03), and from June to July and August (OR 1.2 95%CI 1.0–1.4 *P =* 0.02).

Calves were more likely to die before weaning if born to heifers exposed to breeding in April or earlier as compared to May (OR 1.5 95%CI 1.1–2.2 *P =* 0.02), June (OR 2.0 95%CI 1.4–2.8 p<0.001) or July or August (OR 1.4 95%CI 1.1–1.9 *P =* 0.02). However, calves were also more likely to die before weaning if born to heifers exposed to breeding in July or August as compared to June (OR 1.4 95%CI 1.1–1.9 *P =* 0.01).

Calf death loss from 24 hours to weaning was significantly higher for calves from cows in Alberta than in either Saskatchewan (p<0.001) or Manitoba (p<0.001). Calf death loss from 24 hours to weaning was also significantly higher for calves born to heifers in Alberta (*P =* 0.004) and Manitoba (p<0.001) than in Saskatchewan.

Calf death loss between 24 hours and weaning was significantly lower in 2014 (*P =* 0.047), 2015 (p<0.001), or 2016 (*P =* 0.008) than in 2017 for calves born to cows (Table 4). The death loss in calves from heifers was significantly lower in 2015 than in 2014 (*P =* 0.001), 2016 (p<0.001), and 2017 (*P =* 0.04). It was also lower in 2017 than 2016 (*P =* 0.003).

There was no significant difference in the risk of death between 24 hours and weaning based on whether or not the herd sold any purebred cattle for cows (*P =* 0.30) or for heifers (*P =* 0.18) or whether the herd size at calving was >300 for cows (*P =* 0.83). The risk of death between 24 hours and weaning was higher for calves from heifers from herds with >300 cows at calving (OR 1.8 95%CI 1.4–2.4 p<0.001).

The percentage of total variation in calf loss from birth to weaning of cows attributed to differences between herds was 6.8% and the percentage of total variation attributed to year to year differences within herds for cows was calculated to be 5.8%. The percentage of total variation in calf loss from birth to weaning of heifers attributed to differences between herds was 12% and the percentage of total variation attributed to year to year differences within herds for heifers was calculated to be 9.9%.

## Discussion

This study provides the largest multiyear description of reproductive performance in western Canadian beef herds to date and the first multiyear study of cow-calf performance published since 2001 [13]. The recruitment of herds for this study provided a distribution of herd sizes and geographic location that was representative of the herd distribution in Western Canada, if considering herd sizes of at least 100 cows. However, the recruited herds all had a relationship with a veterinary practice which introduces a selection bias. Herd managers who work routinely with veterinarians, regularly pregnancy test their cows and who volunteer to participate in a research study are most likely better managers and as a result this data may represent better managed herds in Western Canada. Previous studies have estimated that approximately 50% of western Canadian beef producers routinely pregnancy test their cattle [3].

Based on the high frequency of producers reporting no abortions and on previous work, it was very likely there was substantial under-reporting in the percentage of cows that aborted between the end of breeding season and the start of calving [5]. This is not surprising under the extensive management conditions typical for most herds in western Canada. Cows are typically not under close daily scrutiny between pregnancy testing and calving. It is quite possible for cows to abort on pasture without being observed. While producers were asked to report both cows that were observed aborting and cows diagnosed pregnant but failed to calve, it is very likely that some cases were still missed.

The values for the most reliable metrics were then summarized to produce benchmarking indicators. Pregnancy data analysis was restricted to herds that use whole herd pregnancy testing. Herd observations that excluded females destined to be culled were not used in the study as were data from herds that only pregnancy tested some cows or heifers for specific management reasons.

If we look at the 25^th^ percentile of non-pregnancy risk as an achievable value, 5% non-pregnancy rates are realistic goals for both cows and heifers managed similarly to the herds in this study. This compares directly to data collected in 2001 and 2002 from 200 herds where the 25^th^ percentiles were 4.3% and 5.2% [4]. More than 75% of herd observations included pregnancy rates for cows above 90% and more than 50% of herd observations exceed 90% pregnancy rates for heifers. Conversely the 95^th^ percentiles of 13% for cows and 24% for heifers provide suggested upper ends for expected risk of non-pregnancy and the need for further investigation. Data from a 2007 National Animal Health Monitoring study of 2,159 cow-calf herds in the USA documented similar calving percentages of 83.2% in heifers and 92.4% in cows, with much greater variability in the heifer calving percentage [14]. The much higher variation around non-pregnancy risk in heifers versus cows seen in this study is similar to other beef cow-calf studies [15]. This would suggest that there is much wider variability in heifer management and selection programs among western Canadian beef herds than for cow management. However, despite this wider variation in non-pregnancy risk for heifers, heifers had pregnancy rates that were similar to adult cows in approximately 50% of cases.

The decision to use losses within the first 24 hours as compared to a more specific definition of stillbirth was a hold-over from the majority of historical reports for cow calf herds [13,15–20]. This definition is not specific to what is classically considered to be stillbirth as it does include losses of calves born alive but that don’t survive due to mismothering, weakness or birth injuries. It does have some advantages for large herds where the birth is not observed, the dead calf is found later and the herd owner only knows the calf died near the time of birth but not when. A quarter of the herd reports included calf death losses within 24 hours of birth that were < 1% for cows and were 0% for heifers. More than half of producers were able to achieve calf losses within 24 hours of <2% in cows and half the observations reflected losses of just over 2.5% in heifers. However, 5% of reported values were >4.8% for cows and 11.5% for heifers indicating abnormal losses.

The values for calf losses within 24 hours of birth from the current study were similar to those reported in a 2010 survey of 303 cow-calf producers where the median percent of calves dead at or within the first hour of birth was 1.6% (interquartile range (IQR) 0.8% to 2.9%). These numbers are lower than that reported from a large 2002 study of 203 cow-calf herds in western Canada where the median for calves dead within 1 hour of birth was 2.4% (IQR 1.3% to 3.9%) for cows and heifers combined [6]. A large survey from the USA in 2007 reported overall calf mortality of 6.4%; with 44.6% of these losses occurring at birth, 17.4% of mortalities within 24 hours of birth. This would equate to a calf mortality rate within 24 hours of birth of 2.96% [21]. The difference between 2002 and the more recent studies could be due to an actual decrease since that time or due to under-reporting associated with the use of questionnaires in the more recent work vs individual animal records in the older study.

Calf deaths from 24 hours to weaning were not broken down into smaller risk periods in this report as questions on the details of when calves died were not asked every year of the study. Calf loss data were expected to be reasonably complete up to the time cow-calf pairs are moved to summer pasture. However, calf losses on extensive and heavily treed summer pasture could potentially be missed and infrequent losses during the summer season that are identified could miss being recorded in busy operations. Half of the herd reports included losses from birth to weaning that were <2% for both heifers and cows. A quarter of herd reports were just over 1% for cows and were 0% for heifers. Five percent of reported calf losses before weaning were >6.9% for cows and 10.4% for heifers.

A large survey from the USA in 2007 reported overall calf mortality of 6.4%; with 28% of these mortalities occurring after 24 hours of age. This would equate to a 2.4% mortality percentage of calves from 24 hours to weaning [21]. A study of individual records and post mortem results 203 herds from 2002 described a cumulative risk from birth to weaning of 4.0% [8]. A 2010 survey described losses summarized into distinct at risk periods up to 1 month of age: 1 hr to 1 mo (median 0.2%, IQR 0.0% to 1.1%, n = 303), 4 d to 1 mo (0.5%, 0.0% to 1.2%, n = 272), and 1 mo to 3 mo (0.4%, 0.0% to 1.2%, n = 56) [3].

Year-to-year variation was significant for all outcomes of interest with the exception of pregnancy rates in heifers. Year-to-year variation was more apparent for calf losses both at and after birth than for pregnancy rates in this sample. Environmental conditions such as temperature and precipitation have been shown to have a significant effect on calf mortality and these factors could be responsible for some of this year to year variation in productivity outcomes [6, 22]. The only somewhat consistent annual trend in the data was that 2015 was a good year for abortion risk and calf loss from birth to weaning in both cows and heifers and for subsequent pregnancy in cows. However, it was also a bad year for calf losses at birth in cows. The only previous study documenting year-to-year variation in the same group of privately owned beef herds, while based on audited, individual animal data was limited to 8 herds [13].

All outcomes of interest consistently displayed greater variation between herds as compared to year to year differences within herds with the exception of abortion and calf death from birth to 24 hours in cows. This would suggest that for these production traits, management differences between herds plays a greater role in affecting the variation of these traits then do year to year changes within herds that are seen due to climatic conditions. However, the abortion risk and risk of calf death from birth to 24 hours in cows did not follow this pattern and variation between herds was very similar to variation within herds.

There were no consistent patterns of differences among provinces, but what we did observe suggests the potential for significant geographic variability in performance across the study region that needs to be considered in designing surveillance programs and in developing management recommendations. Previous herd productivity studies across large regions of western Canada identified geographic variation through the inclusion of precipitation [6]. Variability in reproductive performance across ecoregions was considered in the analysis in several studies but was not significant in the final models [4–6].

The timing of the start of breeding season was a consistent source of variability among the outcome measures of interest. Cows bred in April or earlier to start calving in late December or January were at increased risk of low pregnancy percentages and higher calf losses at birth, as well as higher calf losses before weaning in both cows and heifers. This is not surprising given the extreme cold and resulting confinement and increased infectious disease risk necessary to manage cold weather calving and the challenges of getting cows pregnant on stored feed. Smaller herds and herds having at least some purebred cattle were more likely to start breeding April or earlier.

However, perhaps the more interesting finding was the significant increase in the risk of non-pregnancy for cows and heifers where first exposure to breeding was not until July or August. In contrast, the risks of calf loss at and after birth in cows first exposed to breeding in July and August were significantly lower than for herds that had earlier breeding seasons. One of the most plausible reasons proposed for lower pregnancy percentages in cows first exposed to breeding later in the summer is the challenge of matching the cow’s nutritional needs early in the breeding season during peak lactation, with the stage of the growing season and pasture quality. Larger herds were more likely than smaller herds to start breeding in July or August. It is important to recognize that although there were differences in non-pregnancy risk for different timing of breeding seasons, the decision on selecting the timing of breeding and calving season is based on a wide variety of economic, marketing and labour issues that may offset any disadvantages of low pregnancy percentages.

Increasing herd size was only a significant risk factor only for pregnancy percentage and calf loss before weaning in heifers. Larger herds could be at an increased risk of biosecurity challenges if there have been recent increases in herd size from purchased animals. One previous report did link increased risk of infectious disease in calves to larger herd sizes [3]. One important factor to consider when evaluating these associations is whether and to what extent herd size influences herd records and the quality of reporting and potential for under-reporting. We did see a few rounded numbers and respondent-described estimates in some of the reports for the largest study herds.

No consistent patterns of differences were observed between herds with only commercial cattle and those that sold at least some purebred animals. However, there were a relatively small number of herds that reported being exclusively purebred in the present study limiting the power to examine the impact of potential management differences in seed stock operations.

In most previous studies we have relied on intensive contact with herd owners and individual animal records to ensure data quality [4–6]. This surveillance program was limited to periodic surveys to collect herd-level records. One of the most important lessons from the first rounds of data collection was the importance of always collecting both complete numerator and denominator data on the same surveys as compared to relying on piecing information together across sequential longitudinal surveys. This is needed so that participants can see the relationship between the numerator and denominator when answering questions to increase their understanding of the questions and accuracy of the answers, and to ensure that both values were available at the time of analysis in case some individual surveys are not returned. Prompt data entry and checking are recommended with immediate follow up with producers regarding missed questions and questions that were apparently misinterpreted. The drawbacks to more intensive contact and the demand for more accurate records is the additional demand on participants of the project, greater costs associated with collecting more detailed data and the likelihood that some producers may be reluctant to maintain participation across multiple years.

Others have described asking producers to report the number of cows calving in the first, second and third cycle of the calving season [23]. We asked for this information in the first year of the study but were not successful at getting useful data from most herds. The most common problem was the difficulty in establishing the start of calving season due to challenges such as the large numbers of breeding management groups in many of the herds with different start dates, the use of AI before the main breeding season, the purchase of bred cows, and the absence of individual animal records or sufficient resources to reconcile these issues. One of the authors has previously described the risk of calving late or more than 63 days after the start of the calving season in a surveillance study [13]; however, that data was derived from detailed individual animal records. While the calving distribution pattern can be a very useful diagnostic tool for individual herds, in large commercial herds that have variable record keeping intensity and highly variable breeding management programs, the calving distribution was not a practical or reliable measure for reproductive surveillance.

## Conclusions

For future studies of reproductive performance in client-owned herds, we suggest focussing effort on the accurate collection of pregnancy testing data, stillbirth and calf loss information. Investigators should work with producers to encourage more complete reporting of abortion losses and consistently reporting calf death losses within meaningful categories for age at death rather than just reporting all losses between 24 hours of age and weaning. While the present study was not based on a random sample of all cow-calf producers, the results are most applicable to cow-calf clients of veterinary practices and the data were comparable to previous reports from western Canada. Future studies should also focus on accurate and specific production information for defined periods of risk for calf loss (i.e. within 24 hours of birth, 24 hours to 1 month) as compared to summarizing all losses from birth to weaning. Reporting bias due to sales and purchases of cattle are less likely to be an issue in defined periods during the calving season as compared to later during and after the breeding season.

The percentiles reported when summarizing individual production metrics can be used by veterinarians and producers to benchmark herd performance, to set realistic goals for improvement and to define thresholds requiring diagnostic investigation. The finding of greater variation among herds in heifer performance than among cows suggests opportunities for improvement in heifer management. The finding that later breeding and calving herds were more at risk for poor pregnancy percentages provides documentation of a problem suspected by producers and veterinarians and will require further investigation to determine the best way to minimize the nutritional challenges of getting cows pregnant later in the growing season.

## Supporting information

S1 File Summary of productivity questions. (PDF)

S2 File PregnancyTestingData. (PDF)

S3 File CalvingOutcomeData. (PDF)

## References

[1] Statistics Canada–The Daily. 2017-05-01 2016 Census of Agriculture. Livestock sector characterized by exits and consolidation [Internet]. Ottawa: Government of Canada [cited 2018 Feb14]. Available from https://www150.statcan.gc.ca/n1/daily-quotidien/170510/dq170510a-eng.htm

[2] Jelinski M, Waldner CL. 2018. Changing demographics of the Canadian cow-calf industry for the period 2011 to 2016. Can Vet J. 2018;59: 1001–4. 30197445 PMC6091134

[3] Waldner C, Jelinski M, McIntyre-Zimmer K. Survey of western Canadian beef producers regarding calfhood diseases, management practices, and veterinary service usage. Can Vet J. 2013;54: 559–64. Available from https://www.ncbi.nlm.nih.gov/pmc/articles/PMC3659451/pdf/cvj_06_559.pdf 24155446 PMC3659451

[4] Waldner CL, Garcia Guerra A. Cow attributes, herd management, and reproductive history events associated with the risk of nonpregnancy in cow-calf herds in Western Canada. Therio. 2013;79: 1083–94. 10.1016/j.theriogenology.2013.02.004 23473871

[5] Waldner C. Cow attributes, herd management, and reproductive history events associated with abortion in cow-calf herds from Western Canada. Therio. 2014;81(6): 840–8. 10.1016/j.theriogenology.2013.12.016 24472651

[6] Waldner C. Cow attributes, herd management and environmental factors associated with the risk of calf death at or within one hour of birth and the risk of dystocia in cow-calf herds in Western Canada. Livest Sci. 2014;63: 126–39. 10.1016/j.livsci.2014.01.032

[7] Elghafghuf A, Stryhn H, Waldner C. A cross-classified and multiple membership Cox model applied to calf mortality data. Prev. Vet. Med. 2014;115(1):29–38. 10.1016/j.prevetmed.2014.03.012 24703248 PMC7114250

[8] Waldner CL, Kennedy RI, Rosengren LB, Pollock CM, Clark EG. Gross postmortem and histologic examination findings from abortion losses and calf mortalities in western Canadian beef herds. Can Vet J. 2010;51: 1227–38. Available from https://www.ncbi.nlm.nih.gov/pmc/articles/PMC2957030/pdf/cvj_11_1227.pdf 21286322 PMC2957030

[9] Gow SP, Waldner CL. Antimicrobial drug use and reason for treatment in 203 western Canadian cow-calf 2 herds during calving season. Prev Vet Med. 2009; 90: 55–65. 10.1016/j.prevetmed.2009.03.010 19376600 PMC7132431

[10] Canadian Beef Industry Fast Facts June 2017 [Internet]. Calgary, Alberta: Canadian Cattlemen’s Association. [cited 2018 Feb14]. Available from http://www.cattle.ca/assets/8562411068/CBIfastfactsENGAug3b-WEB.pdf

[11] Statistics Canada [Internet]. Ottawa: Government of Canada. 2011 Census of Agriculture. [cited 2018 Feb14]. Available from https://www.statcan.gc.ca/eng/ca2011/index

[12] Dohoo I, Martin W, Stryhn H. Methods in epidemiologic research 1st ed. Charlottetown: VER Inc.; 2012.

[13] Waldner C. Monitoring beef cattle productivity as a measure of environmental health. Environ Res Sect A. 2001;86: 94–106. 10.1006/enrs.2001.4239 11386747

[14] USDA. 2009. Beef 2007–08, Part II: Reference of beef cow-calf management practices in the United States, 2007–08 USDA: APHIS: VS, CEAH. Fort Collins, CO #N512.0209.

[15] Ganaba R, Bigra-Poulin M, Belanger D, Couture Y. Description of cow–calf productivity in North western Quebec and path models for calf mortality and growth. Prev Vet Med. 1995;24: 31–42.

[16] Berger PJ, Cubas AC, Koehler KJ, Healey MH. Factors affecting dystocia and early calf mortality in Angus cows and heifers. J Anim Sci. 1992;70: 1775–86. 10.2527/1992.7061775x 1634401

[17] McDermott JJ, Allen OB, Martin SW, Alves DM. Patterns of stillbirth and dystocia in Ontario cow–calf herds. Can J Vet Res. 1992;56: 47–55. 1586893 PMC1263502

[18] Dutil L, Fecteau G, Bouchard E, Dutremblay D, Pare J. A questionnaire on the health, management, and performance of cow–calf herds in Quebec. Can Vet J. 1999;40: 649–56. 10495908 PMC1539859

[19] Nix JM, Spitzer JC, Grimes LW, Burns GL, Plyler BB. A retrospective analysis of factors contributing to calf mortality and dystocia in beef cattle. Therio. 1998;49: 1515–23. 10.1016/s0093-691x(98)00097-1 10732015

[20] Wittum TE, Salman MD, King ME, Mortimer RG, Odde KG, Morris DL. Individual animal and maternal risk factors for morbidity and mortality of neonatal beef calves in Colorado, USA. Prev Vet Med. 1994;19: 1–13.

[21] USDA. 2010. Mortality of calves and cattle on US beef cow-calf operations. APHIS info sheet, May 2010. USDA: APHIS: VS, CEAH. Fort Collins, CO #568.0510.

[22] Azzam SM, Kinder JE, Nielsen MK, Werth LA, Gregory KE, Cundiff LV, Koch RM. Environmental effects on neonatal mortality of beef calves. J Anim Sci. 1993;71: 282–90. 10.2527/1993.712282x 8440645

[23] Larson RL, White BJ. Reproductive systems for North American beef cattle herds. Vet Clin North Am Food Anim Pract. 2016;32(2): 245–66. 10.1016/j.cvfa.2016.01.001 27156223

