# Correction: Identifying performance benchmarks and determinants for reproductive performance and calf survival using a longitudinal field study of cow-calf herds in western Canada

**DOI:** 10.1371/journal.pone.0225401

**Published:** 2019-11-12

**Authors:** Cheryl L. Waldner, Sarah Parker, John R. Campbell

There are errors in Table 3. The data from rows 1 through 4 in columns 5 and 6 were incorrectly duplicated in rows 1 through 4 in columns 1 and 2. Please see the complete, correct Table 3 here.

**Table 3:** Production indices for herds from 2014–2017.

Herd Summary Statistics	% females not pregnant when tested		Abortion Cumulative Incidence		Cumulative incidence of calf death from birth– 24 hours		Cumulative incidence of calf death from 24 hours to weaning	
	Cows	Heifers	Cows	Heifers	Cows	Heifers	Cows	Heifers
Mean (SD)	6.8%	9.7%	0.8%	1.3%	2.1%	3.6%	2.5%	2.9%
Standard deviation	3.4%	8.2%	0.9%	2.3%	1.6%	4.5%	2.4%	3.9%
5^th^ percentile	2.2%	0.0%	0.0%	0.0%	0.0%	0.0%	0.0%	0.0%
25^th^ percentile	4.3%	4.2%	0.0%	0.0%	1.0%	0.0%	1.1%	0.0%
Median	6.2%	8.3%	0.7%	0.0%	1.8%	2.6%	1.9%	1.5%
75^th^ percentile	8.9%	12.9%	1.2%	2.1%	2.8%	5.3%	3.3%	4.6%
95^th^ percentile	12.8%	24.1%	2.6%	5.6%	4.8%	11.5%	6.9%	10.4%
Observations	276	270	353	338	359	345	350	332
Number of herds	94	90	105	104	105	105	105	105
