# Supplementary material for: Identifying performance benchmarks and determinants for reproductive performance and calf survival using a longitudinal field study of cow-calf herds in western Canada
Source: PLoS One. 2019 Jul 18;14(7):e0219901. doi: 10.1371/journal.pone.0219901 (PMC6662034; doi:10.1371/journal.pone.0219901)
Supplement: S1 File — (PDF) [file pone.0219901.s001.pdf]

S1 file. Summary of Productivity Questions.

Survey Questions – Background Information Provided to Producers

- Separate calving groups–Herd is separated into different groups that are expected to begin to calve more than 2 months apart. Answer questions for the majority of the herd.
- Cows (includes heifers that calved the previous year).
- Heifers refers to females born last year being exposed to their first breeding this year.
- Number of females you retained that were open – Females that have been assumed not pregnant or the female was not exposed to breeding but kept in the herd.
- Number of females that you expected to calve – Females confirmed or assumed pregnant at the start of calving season.
- Number of females that actually calved - All the females that gave birth to a full-term calf (whether the calf was born alive, born dead, or died within 24 hours).
- A full-term calf is defined as having a full hair coat and fully erupted teeth.
- Number of females that aborted (observed and probable) - All the females that lost a pregnancy, delivered a pre-term calf AND any females that you expected to calve that did not calve.
- Pre-term calf - any calves with no hair or short fine hair coat and teeth not fully erupted.
- Total number of sick calves (treated/untreated and lived/died) - All calves showing any signs of being sick.
- Total number of females exposed to breeding, either by natural service or artificial insemination (AI) - even if the females do not get pregnant.

Breeding to Weaning Survey

About the *'20XX'* Breeding Season

If your operation has multiple breeding groups that are managed separately and calve at different times during the year (more than two months apart), then answer ALL of the following questions referring to the majority of the herd.

1. Can you provide separate data for cows and heifers for breeding?

- ☐ Yes
- ☐ No, I do not keep separate records for cows and heifers.
  (Answer following questions under 'cows')
- ☐ No, I did not have any heifers

2. Please answer the following.

Answer separately for COWS and HEIFERS, if possible. For this question, "heifers" refers to 20XX-born females exposed to their first breeding in 20XX.

	COWS	HEIFERS
Number of bull(s) used for natural service	__________	__________
Number of total females exposed to any breeding	__________	__________
Number of females exposed only to natural service	__________	__________
Number of females bred at least once using artificial insemination (AI) or embryo transfer (ET)	__________	__________

3. From the start of **this breeding season until the last day of weaning** provide the number of head for each statement below:

Number of baby calves purchased __________

Number of cow-calf pairs purchased __________

Number of cows purchased __________

Number of heifers purchased __________

Number of breeding bulls purchased __________

4. Please provide the following DATES:
(Please write the month in text, e.g., JAN-01.)

	COWS	HEIFERS
Breeding season start date	__________	__________
Breeding season end date	__________	__________

Fall Weaning and Pregnancy Checking

About the Weaned *'20XX'* Born Calves

1. What date did you wean your calves?

Please answer separately for COWS and HEIFERS. For operations with multiple wean dates, please provide the first and last weaning dates. (Please write the month in text, e.g., JAN-01)

	COWS	HEIFERS
Wean date(s)	_________	_________

2. What was the total number of calves alive at weaning?

	COWS	HEIFERS
Number of weaned calves	_________	_________

3. For the following - *Please answer only for those cows that calved on farm at full term*

	COWS	HEIFERS
Number of calves born alive	_________	_________
Number of calves that died *(Between day 1 and weaning)*	_________	_________

About the *'2019'* Bred Females

4. What proportion of your females did you or your veterinarian pregnancy check?

- ☐ All
- ☐ Selected a portion
- ☐ None

5. If you only pregnancy checked a portion of your females, please describe how you chose which ones?

6. What date(s) did you pregnancy check? ___________________________________

7. Please tell us how many females you had pregnancy checked and how many were open.

	COWS	HEIFERS
Number of opens	________	________
Number of checked	________	________

Calving Survey

About the *‘20XX’* Calving Season

1. Do you have separate groups of females that start calving at different times of the year (more than two months apart) within your herd? *(e.g. Spring/fall calving groups)*

- ☐ Yes
- ☐ No

2. Please describe how you divided up the **breeding groups** or check Not Applicable if you do not have separate groups.

- ☐ Age
- ☐ Breed
- ☐ Purebred vs Commercial

☐ Not Applicable

☐ Other: ____

If your operation has multiple breeding groups that are managed separately and calve at different times during the year (more than two months apart), then answer ALL of the following questions referring to the majority of the herd.

3. Can you provide separate calving data for cows and heifers for calving?

☐ Yes

☐ No, I do not keep separate records for cows and heifers.
(Answer following questions under 'cows')

☐ No, I did not have any heifers calving

4. What date was the first, full-term (i.e. not premature) calf born in your calving season?
(Please give us the date of birth of the first full-term calf from a female exposed to breeding in your herd. Please write the month in text e.g., JAN-01)

	COWS	HEIFERS
Date first full-term calf born<br>(*Do not include purchased bred females.*)	__________	__________

5. What date was the last calf born?

	COWS	HEIFERS
Date last full-term calf born<br>(*Do not include purchased bred females.*)	__________	__________

6. Provide the number of head for each statement below:

	COWS	HEIFERS
Number of females you retained that were <u>open</u><br>*(Pregnancy checked open or not exposed to breeding but kept in the herd.)*	__________	__________
Number of females that you <u>expected to calve</u><br>*(Confirmed or assumed pregnant at the start of calving season.)*	__________	__________
Number of females that actually <u>calved</u><br>*(Gave birth to a **full-term calf** whether that calf was born alive, born dead, or died within 24 hours)*	__________	__________
Number of females that <u>aborted</u><br>(*Observed and probable - females that lost a pregnancy, delivered a pre-term calf AND any females that you expected to calve that did not calve.)*	__________	__________

7. Provide the total number of full-term births for each of the following:

	COWS	HEIFERS
Number of **sets** of twins	__________	__________
Number of calves born dead or died within 24 hrs	__________	__________

8. From your last weaning date *(last year- 20XX)* and before turn-out for breeding *(this year- 20XX)*, provide the number of head for each statement below:

Number of baby calves purchased __________

Number of cow-calf pairs purchased __________

Number of cows purchased __________

Number of heifers purchased __________

Number of breeding bulls purchased __________
