# Supplementary material for: Identifying performance benchmarks and determinants for reproductive performance and calf survival using a longitudinal field study of cow-calf herds in western Canada
Source: PLoS One. 2019 Jul 18;14(7):e0219901. doi: 10.1371/journal.pone.0219901 (PMC6662034; doi:10.1371/journal.pone.0219901)
Supplement: S2 File — (PDF) [file pone.0219901.s002.pdf]

S2 file. Pregnancy testing and breeding season data.

ProdID	BreedingYear	CalvingYear	PROVCode	CmrclCode	NmCowChk	NmCowOpen	NmHeifChk	NmHeifOpen	nmBrdCow	nmBrdHf	BrdStartCowCode	BrdStartHfCode	nmFeAICow	nmFeAIHf	PercAICow	PercAIHf
1	2014	2015	1	1	260	15	51	1	265	50	4	4	0	0	0.00	0.00
1	2015	2016	1	1	232	23	48	5	250	48	4	4	0	0	0.00	0.00
1	2016	2017	1	1	255	20	55	5	255	50	4	4	0	0	0.00	0.00
1	2017	2018	1	1	106	9					4					
2	2014	2015	1	2	1110	43	35	1	1096	17	4	4	0	0	0.00	0.00
2	2015	2016	1	2	1020	61	22	1	1020	22	3	4	0	0	0.00	0.00
2	2016	2017	1	2	1155	113	38	3	1155	38	4	4	0	0	0.00	0.00
3	2014	2015	1	1	346	48	83	18	346	73	4	4	0	0	0.00	0.00
3	2015	2016	1	1	331	31	121	12	331	121	4	3	0	0	0.00	0.00
3	2016	2017	1	1	336	30	89	9	336	89	4	3	0	0	0.00	0.00
4	2014	2015	1	1	355	17	172	54	340	175	4	4	50	25	0.15	0.14
4	2015	2016	1	1	460	14	174	27	460	174	4	4	0	0	0.00	0.00
4	2016	2017	1	1	533	39	174	7	533	174	4	4	40	30	0.08	0.17
5	2014	2015	1	1	130	10	30	2	125	30	3	3	0	0	0.00	0.00
5	2015	2016	1	1	133	8	31	2	133	32	3	3	0	0	0.00	0.00
5	2016	2017	1	1	136	4	28	2	136	28	3	3	0	0	0.00	0.00
7	2014	2015	1	1	300	16	68	7	287	67	3	3	0	0	0.00	0.00
7	2015	2016	1	1	314	13	94	5	314	94	3	2	0	0	0.00	0.00
7	2016	2017	1	1	344	16	97	5	344	97	3	2	0	25	0.00	0.26
7	2017	2018	1	1	367	47	96	6	367	96	3	2	0	0	0.00	0.00
9	2015	2016	1	2	261	12	34	1	261	34	1	1	72	0	0.28	0.00
10	2015	2016	2	1	180	15	30	3	180	30	4	4	0	0	0.00	0.00
10	2016	2017	2	1	187	15	30	4	187	30	4	4	0	0	0.00	0.00
11	2014	2015	2	1	277	10	49	2	280	32	3	3	0	0	0.00	0.00
11	2015	2016	2	1	280	23	45	2	283	45	3	3	0	0	0.00	0.00
11	2016	2017	2	1	284	18	54	4	284	55	3	3	0	0	0.00	0.00
11	2017	2018	2	1	280	10	53	3	240	53	1	1	0	0	0.00	0.00
12	2014	2015	1	1	112	10	64	5	108	64	4	3	0	0	0.00	0.00
12	2015	2016	1	1	160	5	50	2	162	50	4	3	0	0	0.00	0.00
12	2017	2018	1	1	180	20	62	7	180	63	4	3	0	0	0.00	0.00
13	2016	2017	3	1	887	114	197	30	880	197	4	4	190	0	0.22	0.00
13	2017	2018	3	1	854	93	132	20	875	121	4	4	185	0	0.21	0.00
15	2016	2017	3	2	219	30	120	7	294	124	1		127	77	0.43	0.62
15	2017	2018	3	2	220	20	130	13	190	130	1	1	90	40	0.47	0.31
16	2014	2015	3	1	225	10	23	1			4	4				
16	2015	2016	3	1	250	10	35	1	250	35	3	3				
16	2016	2017	3	1	250	5	50	2	250	50	4	4	0	0	0.00	0.00
16	2017	2018	3	1	260	5	50	2	260	50	4	4	0	0	0.00	0.00
17	2014	2015	1	1	167	9	23	0	135	21	3	3	0	0	0.00	0.00
17	2015	2016	1	1	134	13	23	1	134	23	3	3	0	0	0.00	0.00
17	2016	2017	1	1	138	8	20	3	138	20	3	3	0	0	0.00	0.00
17	2017	2018	1	1	135	13	15	0	136	15	3	3	0	0	0.00	0.00
18	2014	2015	1	1	229	17	60	3	242	60	2	2	0	0	0.00	0.00
18	2015	2016	1	1	250	12	49	6	271	49	2	2	0	0	0.00	0.00
18	2016	2017	1	1	217	12	59	13	230	59	2	2	0	0	0.00	0.00
18	2017	2018	1	1	235	21	55	9	244	56	2	2	0	0	0.00	0.00
19	2014	2015	3	1	141	5	46	2	155	46	2	2	0	0	0.00	0.00
20	2014	2015	3	1	497	36	110	14	499	120	3	3	0	0	0.00	0.00
20	2015	2016	3	1	518	34	105	20	540	105	3	3	0	0	0.00	0.00

20	2016	2017	3	1	543	42	90	18	539	92	3	3	0	0	0.00	0.00
20	2017	2018	3	1	537	44	125	27	550	126	3	3	0	0	0.00	0.00
21	2014	2015	1	1	148	8	36	1	149	36	2	2	0	0	0.00	0.00
21	2015	2016	1	1	158	13	30	3	160	30	2	4	0	0	0.00	0.00
21	2016	2017	1	1	159	7	28	2	159	28	2	2	0	0	0.00	0.00
21	2017	2018	1	1	161	9	39	1	172	39	2	2	0	0	0.00	0.00
22	2014	2015	2	2	187	16	31	3	204	30	2	2	0	0	0.00	0.00
22	2015	2016	2	2	184	18	38	6	184	38	2	2	0	0	0.00	0.00
22	2016	2017	2	2	179	14	35	7	193	35	2	2	0	0	0.00	0.00
22	2017	2018	2	2	191	20	37	10	191	37	2	2	0	0	0.00	0.00
24	2016	2017	2	1	290	11	56	1	290	55	4	2	0	50	0.00	0.91
24	2017	2018	2	1	295	10	33	0	280	50	4	4	0	0	0.00	0.00
25	2014	2015	2	1	280	13	55	1	288	52	3	3	0	0	0.00	0.00
25	2015	2016	2	1	359	12	68	3	359	65	3	3	0	0	0.00	0.00
25	2016	2017	2	1	331	19	104	1	335	117	3	3	0	0	0.00	0.00
25	2017	2018	2	1	341	9	100	5	340	120	3	3	0	0	0.00	0.00
26	2014	2015	2	1	173	5	32	6	141	32	2	2	0	0	0.00	0.00
26	2015	2016	2	1	58	4	31	5	38	31	2	2	0	0	0.00	0.00
26	2016	2017	2	1	39	4	16	2	39	16	2	2	0	0	0.00	0.00
26	2017	2018	2	1	46	5	10	2	46	10	2	2	0	0	0.00	0.00
30	2015	2016	3	1	234	14	56	21	238	56	4	3	208	56	0.87	1.00
30	2016	2017	3	1	237	21	57	14	237	57	3	3	221	57	0.93	1.00
30	2017	2018	3	1	303	7	60	5	303	60	3	3	199	60	0.66	1.00
32	2014	2015	2	1	171	15	60	16	172	65	3	3	0	0	0.00	0.00
32	2015	2016	2	1	173	28	53	12	189	53	3	3	0	0	0.00	0.00
32	2016	2017	2	1	180	20	61	13	181	63	3	3	0	0	0.00	0.00
32	2017	2018	2	1	178	27	49	14	184	49	3	3	0	0	0.00	0.00
33	2014	2015	2	1	170	16	35	3	160	30	1	1	0	0	0.00	0.00
33	2015	2016	2	1	160	5	40	4	150	40	1	1	0	30	0.00	0.75
33	2016	2017	2	1	187	9	40	2	187	40	1	1	0	38	0.00	0.95
34	2014	2015	2	1	294	15	71	4	285	71	3	3	0	0	0.00	0.00
34	2015	2016	2	1	295	16	61	1	295	61	3	2	0	0	0.00	0.00
36	2015	2016	1	1	1500	120	630	110	1500	630	4	4	0	300	0.00	0.48
38	2014	2015	2	2	300	10	100	5	300	90	1	1	50	50	0.17	0.56
38	2015	2016	2	2	280	10	80	5	250	90	1	1	0	0	0.00	0.00
38	2016	2017	2	2	339	22	108	5			1	1				
38	2017	2018	2	2	360	20	140	3	350	140	1	1	80	90	0.23	0.64
39	2014	2015	1	1	116	10	20	0	123	20	3	3	0	0	0.00	0.00
39	2015	2016	1	1	121	11	21	1	122	21	3	3	0	0	0.00	0.00
39	2016	2017	1	1	117	11	23	2	117	23	3	3	0	0	0.00	0.00
41	2014	2015	2	1	175	15			145	0	1	1	0	0	0.00	
41	2016	2017	2	1	190	12	12	5	190	12	1	1	0	0	0.00	0.00
42	2014	2015	1	1	86	6	12	0	86	12	3	3	0	0	0.00	0.00
42	2015	2016	1	1	91	3	8	1	91	8	3	3				
42	2016	2017	1	1	96	3	20	3	96	20	3	3	0	0	0.00	0.00
42	2017	2018	1	1	98	5	10	1	98	10	3	3	0	0	0.00	0.00
43	2014	2015	1	2	263	23	68	1	269	56	2	2	117	56	0.43	1.00
43	2015	2016	1	2	272	15	70	7	243	80	2	2	88	73	0.36	0.91
43	2017	2018	1	2	334	32	117	19	334	117	2	2	161	84	0.48	0.72
44	2014	2015	2	1	439	28	85	9	414	85	4	4	0	0	0.00	0.00
44	2015	2016	2	1	447	29	86	10	448	86	4	3	0	0	0.00	0.00
44	2016	2017	2	1	448	30	80	6	460	81	4	3	0	0	0.00	0.00
44	2017	2018	2	1	426	26	84	17	430	84	4	3	0	0	0.00	0.00

45	2014	2015	1	1	198	10	36	3	198	36	3	3	0	0	0.00	0.00
45	2015	2016	1	1	196	5	35	3	197	35	3	3	0	0	0.00	0.00
45	2016	2017	1	1	193	12	60	21	193	60	3	3	0	0	0.00	0.00
50	2017	2018	2	2				3	330	40	1	1	120	30	0.36	0.75
51	2015	2016	2	1	171	16	29	1	171	29	3	3	0	0	0.00	0.00
51	2017	2018	2	1	193	14	39	2	193	39	3	3	0	0	0.00	0.00
52	2014	2015	2	1	780	60			691	0	4		0	0	0.00	
52	2015	2016	2	1	565	74	211	22	565	214	4	4	0	0	0.00	0.00
52	2016	2017	2	1	575	40	226	29	605	225	4	4	0	0	0.00	0.00
53	2014	2015	3	1	132	12	10	1	121	14	2	2	0	0	0.00	0.00
53	2015	2016	3	1	150	15	15	1	135	15	1	1	0	0	0.00	0.00
53	2016	2017	3	1	134	1	20	0	134	20	1	1	0	0	0.00	0.00
53	2017	2018	3	1	141	16	15	1	141	15	1	1	0	0	0.00	0.00
54	2014	2015	3	1	324	13			330	0	2		0	0	0.00	
55	2014	2015	2	1	201	13	27	3	190	29	2	2	0	0	0.00	0.00
55	2015	2016	2	1	220	9			220	0	2		0	0	0.00	
55	2016	2017	2	1	225	8	15	0	225		2		0	0	0.00	
57	2014	2015	2	1	86	5	14	3	147	31	1	1	0	0	0.00	0.00
57	2015	2016	2	1	152	14	25	6	152	25	1	1	0	0	0.00	0.00
57	2016	2017	2	1	154	30	31	3	160	31	1	1	0	0	0.00	0.00
57	2017	2018	2	1	162	22	41	2	163	41	1	1	0	0	0.00	0.00
58	2014	2015	2	1	141	15	20	1	120	19	2	2	0	0	0.00	0.00
58	2015	2016	2	1	138	17	24	1	138	24	2	2	0	0	0.00	0.00
58	2016	2017	2	1	143	8	20	1	143	20	2	2	0	0	0.00	0.00
58	2017	2018	2	1	167	9	22	4	154	22	1	1	0	0	0.00	0.00
60	2014	2015	3	1	180	12			173	12	4	4	0	0	0.00	0.00
60	2015	2016	3	1	185	18	12	0	185	12	4	4	0	0	0.00	0.00
62	2014	2015	1	1	469	25	123	15	490	119	4	4	0	0	0.00	0.00
62	2015	2016	1	1	493	45	82	12	498	82	4	4	0	0	0.00	0.00
62	2016	2017	1	1	487	30	330	41	487	330	4	4	0	0	0.00	0.00
63	2014	2015	2	2	81	4	19	2	84	19	1	1	37	8	0.44	0.42
63	2015	2016	2	2	89	2	29	7	89	29	1	1	55	24	0.62	0.83
63	2016	2017	2	2	95	5	30	3	97	30	1	1	37	14	0.38	0.47
63	2017	2018	2	2	111	2	36	3	114	36	1	1	28	18	0.25	0.50
64	2014	2015	1	1	137	5	30	0	138	29	4	3	0	29	0.00	1.00
64	2015	2016	1	1	149	4	28	0	152	29	3	3	60	29	0.39	1.00
64	2016	2017	1	1	162	3	40	2	162	40	3	3	10	40	0.06	1.00
65	2014	2015	2	1	142	4	30	2	142	30	3	3	0	0	0.00	0.00
65	2015	2016	2	1	148	3	22	1	153	22	3	3	0	0	0.00	0.00
65	2016	2017	2	1	165	7	18	2	165	18	3	3	0	0	0.00	0.00
65	2017	2018	2	1	161	8	16	8	159	18	3	3	0	0	0.00	0.00
66	2017	2018	2	2	250	15	46	3	250	46	2	2	36	0	0.14	0.00
67	2014	2015	1	1	175	10	12	0	165	12	4	4	0	0	0.00	0.00
67	2015	2016	1	1	173	14	20	0	174	20	4	4	0	0	0.00	0.00
68	2014	2015	2	1	286	11	35	3	264	35	3	3	0	0	0.00	0.00
68	2015	2016	2	1	322	24	45	5	334	45	4	3	0	0	0.00	0.00
68	2017	2018	2	1	297	23	67	2	306	67	4	3	0	0	0.00	0.00
69	2014	2015	2	2	252	29	53	8	252	54	2	2	58	46	0.23	0.85
69	2015	2016	2	2	234	11	58	9	235	58	2	2	68	49	0.29	0.84
69	2016	2017	2	2	250	17	51	9	250	51	2	2	23	47	0.09	0.92
69	2017	2018	2	2	221	22	52	5	218	52	2	2	21	48	0.10	0.92
71	2014	2015	2	1	153	9	33	1	127	25	2	2	0	0	0.00	0.00
71	2015	2016	2	1	165	8	23	1	158	20	2	2	0	0	0.00	0.00

71	2016	2017	2	1	159	9	21	1	140	20	2	2	0	0	0.00	0.00
71	2017	2018	2	1	148	8	24	1	148	24	1	1	0	0	0.00	0.00
73	2014	2015	2	1	350	45	147	35	350	147	4	4	0	0	0.00	0.00
73	2015	2016	2	1	496	40	151	9	498	151	4	4	0	0	0.00	0.00
73	2016	2017	2	1	810	72	223	40	854	224	4	4	0	0	0.00	0.00
73	2017	2018	2	1	858	95	265	35	869	266	4	4	0	0	0.00	0.00
74	2014	2015	3	1	147	11	12	1	140	22	1	1	0	0	0.00	0.00
74	2015	2016	3	1	140	10	24	0	140	24	1	1	0	0	0.00	0.00
74	2016	2017	3	1	145	7	20	0	145	20	1	1	0	0	0.00	0.00
74	2017	2018	3	1	127	3	25	0	127	23	1	1	0	0	0.00	0.00
75	2014	2015	2	2	2800	150	450	80	2500	500	4	4	0	0	0.00	0.00
75	2015	2016	2	2	2300	90	360	50	2300	360	4	4	0	0	0.00	0.00
77	2014	2015	1	1	188	20	48	1	192	48	1	1	0	0	0.00	0.00
77	2015	2016	1	1	199	11	36	3	200	36	1	1	0	0	0.00	0.00
78	2014	2015	2	1	319	30	155	28	336	154	4	4	8	60	0.02	0.39
78	2015	2016	2	1	315	15	139	15	325	139	4	4	0	54	0.00	0.39
78	2016	2017	2	1	314	15	32	1	450	32	4	4	0	0	0.00	0.00
78	2017	2018	2	1	414	38	30	2	468	102	4	4	0	0	0.00	0.00
80	2014	2015	3	1	165	10	34	0	162	34	2	2	0	0	0.00	0.00
80	2015	2016	3	1	176	10	26	3	177	26	2	2	0	0	0.00	0.00
80	2016	2017	3	1	182	4	26	3	182	26	2	2	4	0	0.02	0.00
80	2017	2018	3	1	185	9	28	1	185	28	2	2	0	0	0.00	0.00
81	2014	2015	2	1	106	5	24	1	101	20	3	3	0	0	0.00	0.00
81	2015	2016	2	1	102	6	23	4	110	23	3	3	0	0	0.00	0.00
81	2016	2017	2	1	101	7	23	1	101	23	3	3	0	0	0.00	0.00
83	2014	2015	2	1	825	48	170	21	750	175	4	2	0	0	0.00	0.00
83	2015	2016	2	1			260	42	794	270	4	2	0	110	0.00	0.41
83	2016	2017	2	1	992	28	180	21	992	180	4	2	0	55	0.00	0.31
83	2017	2018	2	1	850	26	175	30	850	175	4	2	0	120	0.00	0.69
84	2014	2015	2	2	202	17	48	4	209	46	4	4	0	0	0.00	0.00
84	2015	2016	2	2	220	11	36	0	220	36	4	4	0	0	0.00	0.00
84	2016	2017	2	2	237	16	49	6	239	49	4	4	0	0	0.00	0.00
84	2017	2018	2	2	222	15	60	5	224	60	4	4	0	0	0.00	0.00
85	2014	2015	2	2	341	27	60	6	339	56	3	3	24	17	0.07	0.30
85	2015	2016	2	2	436	13	64	4	402	61	1	3	13	29	0.03	0.48
85	2016	2017	2	2	399	21	144	18	399	144	3	3	0	73	0.00	0.51
85	2017	2018	2	2	382	33	108	5	377	125	1	1	0	0	0.00	0.00
86	2014	2015	2	1	1079	116	380	34	1087	393	4	4	0	0	0.00	0.00
86	2015	2016	2	1	1220	95	305	29	1225	305	4	4	0	0	0.00	0.00
86	2016	2017	2	1	1320	95	342	32	1340	350	4	4	0	0	0.00	0.00
86	2017	2018	2	1	1300	109	347	24	1300	347	4	4	0	0	0.00	0.00
87	2016	2017	1	1	198	16	34	1	198	34	3	3	0	0	0.00	0.00
89	2014	2015	2	1	221	7	103	10	225	106	4	4	0	0	0.00	0.00
89	2015	2016	2	1	257	10	72	6	257	72	4	4	0	0	0.00	0.00
89	2016	2017	2	1	280	12	103	8	280	103	4	4	0	0	0.00	0.00
91	2014	2015	3	2	715	36	73	11	683	73	3	2	0	0	0.00	0.00
91	2016	2017	3	2	622	36	96	24	624	96	3	3	0	0	0.00	0.00
91	2017	2018	3	2	482	39	126	13	475	126	1	1	0	0	0.00	0.00
92	2016	2017	1	1	306	28	115	10	325	115	3	3	0	0	0.00	0.00
94	2015	2016	1	2	250	4	70	3	248	70	2	1	45	70	0.18	1.00
94	2017	2018	1	2	243	4	56	1	224	57	1	1	67	57	0.30	1.00
95	2014	2015	3	2	121	13	47	1	119	41	4	4	65	0	0.55	0.00
96	2014	2015	2	1	131	7	18	2	130	18	3	3	0	0	0.00	0.00

96	2015	2016	2	1	131	6	30	3	131	30	3	3	0	0	0.00	0.00
97	2016	2017	1	1	462	56	204	31	370	130	4	4	0	0	0.00	0.00
98	2014	2015	1	1	460	22	150	15	465	150	2	2	0	0	0.00	0.00
98	2015	2016	1	1	470	15	180	16	470	180	2	2	0	0	0.00	0.00
98	2016	2017	1	1	460	15	178	18	460	178	2	2	0	0	0.00	0.00
98	2017	2018	1	1	406	14	62	2	460	170	2	2	0	0	0.00	0.00
99	2014	2015	1	2	110	7	14	1	94	19	4	4	0	0	0.00	0.00
99	2015	2016	1	2	91	3	19	3	102	19	3	3	0	0	0.00	0.00
99	2016	2017	1	2	105	10	15	2	105	15	4	4	0	0	0.00	0.00
99	2017	2018	1	2	108	13	15	1	110	15	4	4	0	0	0.00	0.00
100	2014	2015	1	2	185	5	10	0	175	12	1	1	0	0	0.00	0.00
100	2016	2017	1	2	163	2	9	0	163	9	3	3	0	0	0.00	0.00
100	2017	2018	1	2	155	18	12	0	155	12	3	3	0	0	0.00	0.00
101	2014	2015	2	2	160	7	5	1	160	5	3	3	0	0	0.00	0.00
101	2015	2016	2	2	160	6	31	0	160	31	3	3	0	0	0.00	0.00
101	2016	2017	2	2	198	7	13	0	198	13	1	1	0	0	0.00	0.00
101	2017	2018	2	2	190	3	65	2	190	65	3	3	0	0	0.00	0.00
102	2014	2015	3	1	300	26	31	5	310	25	1	1	0	0	0.00	0.00
102	2015	2016	3	1	280	70	48	25	280	48	1	1	0	0	0.00	0.00
102	2016	2017	3	1	220	22	65	4	220	65	1	1	0	0	0.00	0.00
102	2017	2018	3	1	224	7	42	11	225	42	1	1	0	0	0.00	0.00
104	2014	2015	2	1	268	23	67	10	268	67	1	1	0	0	0.00	0.00
104	2015	2016	2	1	277	19	74	1	277	74	1	1	0	0	0.00	0.00
104	2016	2017	2	1	284	19	93	6	284	93	1	1	0	0	0.00	0.00
104	2017	2018	2	1	275	11	54	7			1	1				
106	2014	2015	2	1	74	4	13	1	63	10	3	3	0	0	0.00	0.00
106	2015	2016	2	1	67	5	10	0	67	10	4	4				
106	2016	2017	2	1	62	3	5	0	62	5	3	3	0	0	0.00	0.00
107	2014	2015	2	2	136	11	38	9	166	39	1	1	106	16	0.64	0.41
107	2015	2016	2	2	146	13	67	3	146	67	1	1	120	20	0.82	0.30
107	2016	2017	2	2	40	5	10	2	40	14	1	1	0	0	0.00	0.00
108	2014	2015	2	1	304	46	60	2	298	60	1	1	0	0	0.00	0.00
108	2015	2016	2	1	311	24	67	5	311	67	1	1	0	0	0.00	0.00
108	2016	2017	2	1	323	35	65	2	323	65	1	1	0	0	0.00	0.00
110	2014	2015	2	2	115	4	33	3	150	26	1	1	4	0	0.03	0.00
110	2015	2016	2	2	145	6	26	1	145	24	1	1	4	0	0.03	0.00
110	2016	2017	2	2	124	8	29	3	116	26	1	1	10	0	0.09	0.00
110	2017	2018	2	2	132	7	30	0	140	40	1	1	10	10	0.07	0.25
111	2015	2016	3	2	131	16	33	4	131	33	4	4	0	0	0.00	0.00
111	2016	2017	3	2	141	15	32	3	141	32	4	4	0	0	0.00	0.00
111	2017	2018	3	2	65	3	39	1	100	40	4	4	0	0	0.00	0.00
112	2014	2015	1	1	198	18	48	0	200	48	4	4	0	0	0.00	0.00
112	2015	2016	1	1	352	14	69	4	355	69	4	4	0	0	0.00	0.00
112	2016	2017	1	1	375	31			376	0	4		0	0	0.00	
112	2017	2018	1	1	324	24	51	4	329	51	4	4	0	0	0.00	0.00
113	2015	2016	3	1	103	2	12	2	103	12	2	2	0	0	0.00	0.00
113	2016	2017	3	1	99	11	14	2	110	14	3	3	0	0	0.00	0.00
113	2017	2018	3	1	81	8	8	1	92	14	3	3	0	0	0.00	0.00
114	2014	2015	1	1	363	24	63	6	362	71	4	3	0	0	0.00	0.00
114	2015	2016	1	1	365	21	145	19	365	146	4	3	0	0	0.00	0.00
115	2015	2016	2	2	137	17	52	2	137	52	4	4	89	52	0.65	1.00
118	2015	2016	2	1	110	17	51	2	100	50	4	3	0	0	0.00	0.00
118	2016	2017	2	1	134	8	56	6	128	56	4	3	0	0	0.00	0.00

118	2017	2018	2	1	164	17	50	3	164	50	4	3	0	0	0.00	0.00
119	2015	2016	2	1	458	41	171	34	475	172	4	4	0	0	0.00	0.00
119	2016	2017	2	1	476	45	145	27	415	130	4	4	0	0	0.00	0.00
120	2015	2016	2	1	198	6	26	0	198	26	1	1	0	0	0.00	0.00
120	2016	2017	2	1	203	8	22	1	206	22	1	1	0	0	0.00	0.00
120	2017	2018	2	1	203	15	24	1	205	24	1	1	0	0	0.00	0.00
121	2016	2017	2	1	275	10	44	2	276	44	2	2	0	0	0.00	0.00
121	2017	2018	2	1	293	11	58	9	295	58	2	2	0	0	0.00	0.00
123	2017	2018	1	1	137	8	30	0	137	30	2	2	0	0	0.00	0.00
124	2017	2018	1	1	370	12	90	11	360	100	3	3	0	0	0.00	0.00
125	2016	2017	1	1	165	10	25	0	171	28	4	4	6	2	0.04	0.07
125	2017	2018	1	1	229	21	34	3	160	35	4	4	5	3	0.03	0.09
128	2016	2017	3	1	193	31	36	4	193	36	1	1	0	0	0.00	0.00
128	2017	2018	3	1	181	29	39	10	178	41	1	1	0	0	0.00	0.00
129	2015	2016	1	1	316	8	46	3	316	46	4	3				
129	2016	2017	1	1	285	4	51	2	285	51	4	3	0	0	0.00	0.00
129	2017	2018	1	1	282	3	53	4	310	53	4	3	0	0	0.00	0.00
