# Supplementary material for: Identifying performance benchmarks and determinants for reproductive performance and calf survival using a longitudinal field study of cow-calf herds in western Canada
Source: PLoS One. 2019 Jul 18;14(7):e0219901. doi: 10.1371/journal.pone.0219901 (PMC6662034; doi:10.1371/journal.pone.0219901)
Supplement: S3 File — (PDF) [file pone.0219901.s003.pdf]

S3 file. Calving season data.

ProdID	BreedingYear	CalvingYear	PROVCode	CmrclCode	nmClvdCow	nmClvdHf	CLVDGR300	CLVDGR500	nmAbrtCow	denAbrtCow	nmAbrtHf	denAbrtHf	nmTwinCow	nmTwinHf	CALVESBORNCOWS	nmClvBrnDdCow	CALVESBORNHF	nmClvBrnDdHf	denClvDdWnCow	nmClvDdWnCow	denClvDdWnHf	nmClvDdWnHf	nmWnClvCow	nmWnClvHf	BrdStartCowCode	BrdStartHfCode
1	2013	2014	1	1	285		0	0	2	287	0		1	0	286	7			279	6			266		4	4
2	2013	2014	1	2	974	25	1	1	7	981	2	27	0	0	974	13	25	1	961	25	24	3	810	20	4	4
3	2013	2014	1	1	168	32	0	0	0	168	1	33	2	0	170	3	32	1	167	0	31	0	165	31	4	4
4	2013	2014	1	1	382	120	1	1	3	385	2	122	2	0	384	6	120	4	378	16	116	6	243	111	4	4
5	2013	2014	1	1	101	27	0	0	3	104	1	28	3	1	104	4	28	3	100	1	25	0	100	27	3	3
7	2013	2014	1	1	263	59	1	0	1	264	1	60	6	1	269	7	60	6	262	7	54	4	261	49	3	3
8	2013	2014	1	2																					1	1
9	2013	2014	1	2	237	26	0	0	1	238	3	29	9	0	246	11	26	3	235	10	23	3	216	21		
10	2013	2014	2	1																					4	4
11	2013	2014	2	1	285	17	1	0	3	288	0	17	3	0	288	3	17	1	285	13	16	0	269	15	3	3
12	2013	2014	1	1	134	15	0	0	0	134	0	15	2	0	136	4	15	2	132	2	13	0	128	13	4	4
13	2013	2014	3	1																					4	4
14	2013	2014	3	1	135	27	0	0	0	135	0	27	0	0	135	1	27	1	134	2	26	0	133	26	4	4
15	2013	2014	3	2	163	49	0	0	1	164	2	51	7	2	170	8	51	4	162	3	47	3				
16	2013	2014	3	1	220	33	0	0	2	222	0	33	5	1	225	0	34	0	225	1	34	0	225	33	4	4
17	2013	2014	1	1	109	16	0	0	0	109	1	17	0	0	109	3	16	1	106	1	15	0	105	15	3	3
18	2013	2014	1	1	203	49	0	0	0	203	1	50	8	0	211	4	49	3	207	5	46	0	202	46	3	3
19	2013	2014	3	1	121	24	0	0	1	122	1	25	3	0	124	3	24	1	121	9	23	1	111	23	2	2
20	2013	2014	3	1	389	86	1	0	3	392	1	87	6	0	395	3	86	3	392	5	83	3	380	84	3	3
21	2013	2014	1	1	131	29	0	0	1	132	0	29	3	0	134	3	29	3	131	1	26	0	129	27	2	2
22	2013	2014	2	2	166	26	0	0	0	166	1	27	3	0	169	3	26	0	166	2	26	0	166	26	2	2
23	2013	2014	2	2	750	120	1	1	5	755	3	123	6	0	756	6	120	6	750	6	114	2	715	115	4	4
25	2013	2014	2	1	255	55	1	0	1	256	0	55	4	1	259	8	56	0	251	2	56	0	248	54	3	3
26	2013	2014	2	1	119	28	0	0	3	122	0	28	3	0	122	3	28	3	119	2	25	0	113	25	2	2
29	2013	2014	2	1																					3	3
30	2013	2014	3	1	211	34	0	0	2	213	1	35	3	0	214	6	34	0	208	2	34	0	203	33	4	4
32	2013	2014	2	1	158	36	0	0	4	162	0	36	0	0	158	1	36	3	157	2	33	0	148	19	3	3
33	2013	2014	2	1	140	33	0	0	2	142	0	33	5	2	145	4	35	2	141	4	33	3	135	30	1	1
34	2013	2014	2	1	270	50	1	0	2	272	1	51	6	0	276	12	50	5	264	3	45	1	246	48	3	3
36	2013	2014	1	1																					4	4
37	2013	2014	2	1																					3	3
38	2013	2014	2	2	226	56	0	0	0	226	0	56	6	0	232	4	56	6	228	7	50	0	200	50	1	1
39	2013	2014	1	1	96	34	0	0	0	96	0	34	2	1	98	3	35	2	95	4	33	0	96	34	3	3
40	2013	2014	2	2																					1	1
41	2013	2014	2	1	170		0	0	2	172	0		2	0	172	0			172	2			170		1	1
42	2013	2014	1	1	77	7	0	0	1	78	1	8	0	0	77	2	7	0	75	0	7	0	78	6	3	3
43	2013	2014	1	2	259	27	0	0	4	263	0	27	15	1	274	4	28	0	270	4	28	1	234	26	2	2
44	2013	2014	2	1	360	69	1	0	4	364	3	72	6	1	366	4	70	5	362	2	65	0	356	62	4	4
45	2013	2014	1	1	299	63	1	0	0	299	1	64	4	0	303	3	63	1	300	1	62	0			3	3
46	2013	2014	2	1	300	37	1	0	1	301	0	37	12	3	312	18	40	1	294	7	39	0	289	36	2	2
48	2013	2014	1	1																						
50	2013	2014	2	2	348	36	1	0	2	350	1	37	13	2	361	3	38	1	358	16	37	1		33		
51	2013	2014	2	1	158		0	0	1	159	0	1	0	0	158	2	1	0	156	1			174	1	3	3
52	2013	2014	2	1	725		1	1	0	725	0		2	0	727	20		0	707	16			690	0		
53	2013	2014	3	1	115	20	0	0	0	115	0	20	2	0	117	3	20	0	114	2	20	1		20	1	1
54	2013	2014	3	1	246		0	0	1	247			9		255	5			250	21			319		1	1
55	2013	2014	2	1	185	35	0	0	5	190	0	35	8	0	193	4	35	2	189	13	33	1	178	33	2	2
56	2013	2014	2	1																					4	4
57	2013	2014	2	1	122	29	0	0	2	124	0	29	7	0	129	5	29	2	124	0	27	0	122	29	1	1
58	2013	2014	2	1									0	0											2	2
59	2013	2014	2	1																					3	3
60	2013	2014	3	1	200	10	0	0	0	200	0	10	0	0	200	8	10	4	192	2	6	0	190	6	4	4
61	2013	2014	2	1	87	41	0	0	0	87	1	42	1	0	88	2	41	4	86	1	37	0	84	36	3	3
62	2013	2014	1	1	421	90	1	1	3	424	0	90	5	1	426	5	91	6	421	3	85	3	408	81	4	4
63	2013	2014	2	2	66	17	0	0	2	68	1	18	3	1	69	5	18	1	64	2	17	1	60	19	1	1
64	2013	2014	1	1									0	0	133	1	2	2	132	1			131		4	4
65	2013	2014	2	1	133	27	0	0	2	135	0	27	3	1	136	3	28	0	133	0	28	1	114	26	3	3
66	2013	2014	2	2	211	36	0	0	3	214	1	37	7	0	218	5	36	0	213	1	36	0	206	33	2	2
67	2013	2014	1	1	158	12	0	0	3	161	0	12	1	0	159	2	12	1	157	3	11	0	154	7	4	4
68	2013	2014	2	1	296	26	1	0	2	298	0	26	4	0	300	13	26	4	287	24	22	2	259	21	3	3
69	2013	2014	2	2	218	40	0	0	1	219	0	40	11	0	229	3	40	0	226	5	40	0	214	39	2	2
71	2013	2014	2	1	143	21	0	0	3	146	0	21	8	0	151	3	21	2	148	6	19	2	131	17	2	2
72	2013	2014	3	1																					4	4
73	2013	2014	2	1	268	91	1	0	16	284	4	95	10	1	278	7	92	1	271	9	91	1	262	90	4	4
74	2013	2014	3	1	157	20	0	0	1	158	0	20	4	0	161	5	20	0	156	1	20	0	155	20	1	1
75	2013	2014	2	2	2350	180	1	1	50	2400	2	182	50	2	2400	50	182	5	2350	50	177	16	2270	160		
77	2013	2014	1	1	147	50	0	0	1	148	0	50	11	0	158	5	50	3	153	2	47	0	149	48	1	1
78	2013	2014	2	1	311	79	1	0	9	320	3	82	8	0	319	3	79	2	316	10	77	8	275	55	4	4
80	2013	2014	3	1	146	16	0	0	4	150	0	16	6	0	152	1	16	0	151	6	16	1	145	16	2	2
81	2013	2014	2	1	95	15	0	0	0	95	0	15	1	0	96	1	15	0	95	2	15	0	90	15	3	3
83	2013	2014	2	1	750	165	1	1	5	755	1	166	11	3	761	11	168	4	750	25	164	30	720	133	4	4
84	2013	2014	2	2	152	38	0	0	0	152	0	38	0	1	152	1	39	1	151	4	38	3	151	36	4	4
85	2013	2014	2	2	330	35	1	0	3	333	1	36	12	0	342	6	35	1	336	12	34	1	321	32	3	3
86	2013	2014	2	1	913	218	1	1	9	922	2	220	10	3	923	17	221	9	906	18	212	6	869	201	4	4
87	2013	2014	1	1	145	33	0	0	0	145	0	33	2	0	147	3	33	2	144	3	31	1	81	19	3	3
88	2013	2014	1	1																					2	2
89	2013	2014	2	1	169	66	0	0	0	169	0	66	2	1	171	7	67	0	164	2	67	4	160	62	4	4
90	2013	2014	2	1	147	56	0	0	1	148	0	56	4	2	151	3	58	6	148	2	52	0	145	50		
91	2013	2014	3	2	755	43	1	1	7	762	1	44	23	1	778	38	44	2	740	23	42	1	717	40	3	3
92	2013	2014	1	1	250	71	1	0	3	253	1	72	6	0	256	3	71	4	253	2	67	1	250	66	3	3
94	2013	2014	1	2	205	43	0	0	2	207	0	43	4	0	209	3	43	3	206	6	40	1	200	38	2	2
95	2013	2014	3	2	112	20	0	0	0	112	2	22	5	0	117	7	20	0	110	0	20	0	109	20	1	1
96	2013	2014	2	1	118	18	0	0	0	118	0	18	1	0	119	3	18	0	116	2	18	0	114	15	3	3
97	2013	2014	1	1	300	100	1	0	3	303	1	101	0	0	300	0	100	0	300	2	100	1	300	100	4	4
98	2013	2014	1	1																					2	2
99	2013	2014	1	2	86	12	0	0	0	86	0	12	0	0	86	3	12	0	83	1	12	0	82	12	4	4
100	2013	2014	1	2	142	25	0	0	1	143	0	25	5	0	147	10	25	0	137	5	25	0	129	25	3	3
101	2013	2014	2	2	148	25	0	0	2	150	0	25	1	0	149	4	25	0	145	6	25	2	138	23	3	3
102	2013	2014	3	1	260	60	1	0	7	267	1	61	10	2	270	5	62	1	265	40	61	5	227	53		
103	2013	2014	2	2																					3	3
104	2013	2014	2	1	217	47	0	0	0	217	1	48	4	1	221	2	48	0	219	15	48	8	192	37	1	1
106	2013	2014	2	1	60	14	0	0	1	61	0	14	2	0	62	2	14	0	60	5	14	0	52	14		
107	2013	2014	2	2	128	33	0	0	2	130	0	33	8	1	136	3	34	1	133	5	33	0	115	33	1	1
108	2013	2014	2	1	252	52	1	0	1	253	1	53	11	2	263	9	54	2	254	5	52	1	249	51	1	1
110	2013	2014	2	2	103	33	0	0	2	105	0	33	0	0	103	0	33	0	103	6	33	2	97	31	1	1
111		2014	3	1	94	38	0	0	0	94	0	38	1	2	95	8	40	6	87	4	34	4	82	28		
112		2014	1	1	180	38	0	0	3	183	2	40	3	0	183	2	38	1	181	0	37	0	175	38		
113		2014	3	1																						
114		2014	1	1	280	89	1	0	4	284	1	90	4	0	284	5	89	2	279	1	87	1	273	88		
115		2014	2	2	113	25	0	0	3	116	0	25	0	0	113	4	25	1	109	16	24	5	94	20		
1	2014	2015	1	1	204	49	0	0	2	206	0	49	4	0	208	5	49	1	203	5	48	0	194	49	4	4
2	2014	2015	1	2	1081	17	1	1	15	1096	0	17			1046	24	16	0	1022	5	16	0	1017	16	4	4
3	2014	2015	1	1	297	63	1	0	0	297	1	64	3	0	300	4	63	2	296	2	61	1	295	62	4	4
4	2014	2015	1	1	338	174	1	1	1	339	1	175	3	1	341	9	175	4	332	12	171	3	314	166	4	4
5	2014	2015	1	1	111	26	0	0	1	112	1	27	1	0	112	0	26	0	112	3	26	0	109	25	3	3

7	2014	2015	1	1	269	61	1	0	2	271	0	61	4	0	273	2	61	3	271	8	58	2	276	54	3	3
9	2014	2015	1	2	245	18	0	0	0	245	0	18	5	0	250	3	18	1	247	4	17	0	243	17	2	2
10	2014	2015	2	1	156	34	0	0	0	156	0	34	0	0	156	1	34	1	155	3	33	0	153	34	4	4
11	2014	2015	2	1	268	47	1	0	1	269	0	47	3	1	271	8	48	2	263	9	46	2	248	44	3	3
12	2014	2015	1	1	96	58	0	0	1	97	0	58	1	0	97	1	58	3	96	1	55	0	95	55	4	3
14	2014	2015	3	1	141	27	0	0	0	141	0	27	1	0	142	2	27	2	140	3	25	1	136	24	3	3
15	2014	2015	3	2	180	32	0	0	2	182	2	34	13	0	193	2	32	2	191	1	30	3	179	29	1	1
16	2014	2015	3	1	250	35	0	0	0	250	0	35	7	1	257	5	36	0	252	0	36	0	250	35	4	4
17	2014	2015	1	1	118	20	0	0	0	118	0	20	1	0	119	1	20	1	118	1	19	0	117	19	3	3
18	2014	2015	1	1	210	81	0	0	2	212	0	81	9	2	219	11	83	1	208	1	82	1	207	81	2	2
19	2014	2015	3	1	124	43	0	0	2	126	1	44	6	0	130	6	43	1	124	2	42	2	120	42	2	2
20	2014	2015	3	1	442	93	1	1	4	446	2	95	7	0	449	8	93	2	441	9	91	2	427	89	3	3
21	2014	2015	1	1	138	34	0	0	0	138	0	34	5	1	143	1	35	1	142	2	34	1	95	78	2	2
22	2014	2015	2	2	159	27	0	0	2	161	0	27	2	0	161	3	27	0	158	6	27	0		27	2	2
23	2014	2015	2	2	700	100	1	1	6	706	2	102	10	0	710	6	100	2	704	6	98	2			4	4
25	2014	2015	2	1	292	52	1	0	6	298	0	52	4	1	296	5	53	0	291	8	53	0	284	52	3	3
26	2014	2015	2	1	59		0	0	0	59	0	1	2	0	61	2	1	0	59	0	1	0	57	1	2	2
29	2014	2015	2	1	490	27	1	1	2	492	0	27	4	0	494	6	27	1	488	25	26	2			1	1
30	2014	2015	3	1	215	27	0	0	2	217	0	27	1	0	216	6	27	2	210	4	25	0	203	26	4	4
32	2014	2015	2	1	150	44	0	0	0	150	0	44	1	0	151	2	44	3	149	6	41	2	141	39	3	3
33	2014	2015	2	1	140	28	0	0	0	140	0	28	6	1	146	6	29	3	140	8	26	2	120	27	1	1
34	2014	2015	2	1	283	51	1	0	2	285	0	51	9	0	292	5	51	2	287	7	49	1	264	51	3	3
36	2014	2015	1	1	950	240	1	1	5	955	2	242	8	2	958	25	242	12	933	11	230	6			4	4
38	2014	2015	2	2	300	40	1	0	0	300	1	41	3	0	303	9	40	8	294	5	32	3	283	29	1	1
39	2014	2015	1	1	107	22	0	0	1	108	0	22	5	0	112	1	22	0	111	0	22	0	103	22	3	3
41	2014	2015	2	1	200		0	0	0	200			3		203	2			201	4			194		1	1
42	2014	2015	1	1	81	12	0	0	0	81	0	12	1	0	82	1	12	0	81	0	12	0	78	12	3	3
43	2014	2015	1	2	231	45	0	0	2	233	0	45	15	0	246	8	45	3	238	5	42	3	232	39	2	2
44	2014	2015	2	1	379	76	1	0	2	381	0	76	3	1	382	4	77	2	378	5	75	2	368	72	4	4
45	2014	2015	1	1	165	33	0	0	2	167	0	33	0	0	165	4	33	0	161	2	33	0	164	33	3	3
46	2014	2015	2	1	305	40	1	0	0	305	0	40	14	2	319	18	42	0	301	8	42	0	295	40	2	2
50	2014	2015	2	2	275	38	1	0	3	278	3	41	9	2	284	4	40	2	280	14	38	2	275	27	1	1
51	2014	2015	2	1	154	22	0	0	1	155	0	22	7	0	161	5	22	0	156	0	22	0	151	21	3	3
52	2014	2015	2	1	617		1	1	0	617			1		618	20			598	35			562		4	
53	2014	2015	3	1	121	14	0	0	0	121	0	14	7	1	128	3	15	1	125	1	14	0	124	14	2	2
54	2014	2015	3	1	302		1	0	4	306			11		313	4			309	17			288		2	
55	2014	2015	2	1	187	29	0	0	3	190	1	30	5	0	192	3	29	1	189	13	28	0	176	28	2	2
57	2014	2015	2	1	128	27	0	0	3	131	1	28	15	1	143	5	28	0	138	3	28	0	131	28	1	1
58	2014	2015	2	1	112	19	0	0	0	112	0	19	8	0	120	4	19	1	116	5	18	0	119	18	2	2
60	2014	2015	3	1	183	12	0	0	1	184	0	12	1	0	184	3	12	1	181	0	11	0	181	10	4	4
61	2014	2015	2	1	115	5	0	0	1	116	0	5	1	0	116	0	5	0	116	0	5	0	115	5	3	3
62	2014	2015	1	1	462	108	1	1	3	465	0	108	9	1	471	20	109	8	451	7	101	3	442	99	4	4
63	2014	2015	2	2	77	14	0	0	0	77	0	14	3	0	80	6	14	1	74	5	13	1	69	12	1	1
64	2014	2015	1	1	125	28	0	0	1	126	0	28	2	1	127	3	29	2	124	3	27	0	121	27	4	3
65	2014	2015	2	1	136	26	0	0	1	137	0	26	5	0	141	3	26	1	138	2	25	0	129	25	3	3
66	2014	2015	2	2	199	38	0	0	3	202	0	38	8	0	207	2	38	0	205	4	38	1	200	37	2	2
67	2014	2015	1	1	155	28	0	0	1	156	2	30	1	0	156	3	28	2	153	3	26	1	150	25	4	4
68	2014	2015	2	1	336	32	1	0	2	338	0	32	2	0	338	13	32	0	325	8	32	1	313	31	3	3
69	2014	2015	2	2	193	45	0	0	0	193	0	45	16	2	209	3	47	3	206	4	44	1	193	44	2	2
71	2014	2015	2	1	127	25	0	0	2	129	0	25	7		134	2	25	2	132	3	23	1	122	22	2	2
73	2014	2015	2	1	296	173	1	0	1	297	0	173	4	0	300	4	173	5	296	4	168	1	292	168	4	4
74	2014	2015	3	1	137	20	0	0	2	139	0	20	4	0	141	3	20	0	138	1	20	0	136	20	1	1
75	2014	2015	2	2	2300	400	1	1	20	2320	5	405	50	5	2350	20	405	10	2330	40	395	10	2200	360	4	4
77	2014	2015	1	1	164	45	0	0	2	166	0	45	5	1	169	3	46	2	166	5	44	0	156	43	1	1
78	2014	2015	2	1	275	55	1	0	5	280	2	57	3	0	278	2	55	0	276	1	55	0	272	55	4	4
80	2014	2015	3	1	150	31	0	0	2	152	3	34	5	1	155	0	32	1	155	4	31	1	142	31	2	2
81	2014	2015	2	1	91	19	0	0	0	91	0	19	3	0	94	2	19	0	92	1	19	0	91	19	3	3
83	2014	2015	2	1	683	146	1	1	5	688	2	148	10	3	693	11	149	5	682	16	144	12	662	132	4	2
84	2014	2015	2	2	184	42	0	0	0	184	0	42	6	0	190	2	42	1	188	8	41	0	181	40	4	4
85	2014	2015	2	2	372	75	1	0	7	379	2	77	4	1	376	3	76	0	373	17	76	7	338	69	3	3
86	2014	2015	2	1	941	346	1	1	6	947	2	348	13	5	954	24	351	14	930	22	337	10	879	316	4	4
87	2014	2015	1	1	146	25	0	0	0	146	0	25	2	0	148	3	25	2	145	3	23	0			3	3
89	2014	2015	2	1	209	56	0	0	0	209	1	57	4	0	213	3	56	3	210	1	53	1			4	4
90	2014	2015	2	1	145	26	0	0	2	147	0	26	6	1	151	7	27	0	144	2	27	0	126	24	2	2
91	2014	2015	3	2	545	61	1	1	7	552	1	62	23	0	568	18	61	0	550	7	61	3	543	58	3	2
92	2014	2015	1	1	267	65	1	0	2	269	0	65	7	0	274	4	65	3	270	3	62	0	257	64	3	3
94	2014	2015	1	2	210	38	0	0	1	211	0	38	4	0	214	1	38	0	213	9	38	1	209	36	2	1
95	2014	2015	3	2	107	36	0	0	1	108	1	37	3	0	110	1	36	1	109	1	35	0			4	4
96	2014	2015	2	1	124	15	0	0	0	124	0	15	1	0	125	2	15	3	123	2	12	0	124	12	3	3
97	2014	2015	1	1	336	178	1	1	0	336	0	178	0	0	336	0	178	0	336	0	178	0	336	178	4	4
98	2014	2015	1	1	412	60	1	0	1	413	0	60	4	0	416	2	60	0	414	10	60	2	400	59	2	2
99	2014	2015	1	2	86	13	0	0	0	86	0	13	0	0	86	1	13	0	85	1	13	0	84	13	4	4
100	2014	2015	1	2	169	12	0	0	0	169	0	12	6	0	175	3	12	2	172	3	10	0	167	12	1	1
101	2014	2015	2	2	152	17	0	0	2	154	0	17	3	1	155	4	18	0	151	6	18	0	140	17	3	3
102	2014	2015	3	1	280	20	0	0	5	285	1	21	20	0	300	3	20	2	297	3	18	30	257	20	1	1
104	2014	2015	2	1	244	54	0	0	0	244	0	54	2	0	246	4	54	4	242	18	50	4	225	45	1	1
106	2014	2015	2	1	63	10	0	0	2	65	0	10	3	0	66	3	10	0	63	6	10	0	55	10	3	3
107	2014	2015	2	2	118	33	0	0	3	121	2	35	3	2	121	3	35	1	118	4	34	0	118	33	1	1
108	2014	2015	2	1	255	58	1	0	2	257	0	58	11	0	266	6	58	1	260	2	57	1	254	56	1	1
110	2014	2015	2	2	120	28	0	0	3	123	1	29	3	1	123	0	29	0	123	11	29	3	109	25	1	1
111	2014	2015	3	1	98	22	0	0	1	99	0	22	5	2	103	2	24	4	101	2	20	1	96	19	4	4
112	2014	2015	1	1	172	47	0	0	2	174	0	47	2	0	174	6	47	0	168	2	47	0	168	47	4	4
113	2014	2015	3	1	93	15	0	0	0	93	0	15	3	0	96	4	15	0	92	5	15	0	89	13	2	2
114	2014	2015	1	1	323	62	1	0	0	323	0	62	4	0	327	0	62	2	327	4	60	1	322	61	4	3
115	2014	2015	2	2	125	35	0	0	0	125	0	35	2	0	127	5	35	0	122	5	35	0	116	35	4	4
117		2015	2	2	303	53	1	0	3	306	2	55	15	0	318	9	53	4	309	10	49	1	226	34		
118		2015	2	1	100	30	0	0					2	1	102	2	31	1	100	2	30	1				
119		2015	2	1	358	131	1	0	1	359	1	132	2	1	360	1	132	6	359	6	126	4	344	121		
120		2015	2	1	181	24	0	0	0	181	0	24	6	0	187	4	24	1	183	9	23	0	173	24		
1	2015	2016	1	1											205	2	42	0	203	1	42	2	191	40	4	4
2	2015	2016	1	2																			841	14	3	4
3	2015	2016	1	1	307	79	1	0	2	309	1	80	5	0	305	8	78	1	297	2	77	2	296	69	4	3
4	2015	2016	1	1	427	92	1	1	5	432	3	95	3	1	427	10	92	5	427	35	92	17	332	110	4	4
5	2015	2016	1	1	114	30	0	0			1	31	0	0	114	1	29	0	113	2	29	1			3	3
7	2015	2016	1	1	300	89	1	0	0	300	2	91	7	1	300	2	89	1	298	9	89	0	285	84	3	2
8		2016	1	2																						
9	2015	2016	1	2	291	33	1	0	4	295	0	33	16	1	303	9	34	2	296	5	32	2			1	1
10	2015	2016	2	1	168	29	0	0	2	170	1	30	4	0	168	2	30	0	166	3	29	2	183	26	4	4
11	2015	2016	2	1	257	44	1	0	2	259	1	45	4	0	257	3	44	1	250	4	42	1	249	42	3	3
12	2015	2016	1	1	135	38	0	0	1	136	0	38	3	0	135	2	38	1	133	3	37	0	135	37	4	3
13		2016	3	1					5	639	1	143			634	4	142	2	623	0	139	0	670	135		
14	2015	2016	3	1	140	18	0	0	2	142	0	18	0	0	138	3	18	0	135	1	18	0	131	18	3	3
15	2015	2016	3	2	224	45	0	0	0	224	1	46	6	0	168	1	44	2	173	2	41	2	134	48	1	1
16	2015	2016	3	1	225	35	0	0	0	225	0	35	5	1	225	0	35	0	225	3	35	1	250	40	3	3
17	2015	2016	1	1	133	20	0	0	1	134	0	20	2	0	114	4	20	0	109	2	20	0	108	20	3	3
18	2015	2016	1	1	192	38	0	0	4	196	2	40	6	0	198	6	38	0	192	7	38	0	189	36	2	2
19	2015	2016	3	1	139	47	0	0	1	140	0	47	4	2	142	3	48	4	137	3	47	2	143	44	2	2
20	2015	2016	3	1	489	85	1	1	6	495	1	86	7	2	483	6	85	3	479	15	83	1	459	81	3	3
21	2015	2016	1	1	146	27	0	0	0	146	1	28	1	3	147	1	30	1	139	2	30	0			2	4
22	2015	2016	2	2	161	31	0	0	2	163	0	31	7	0	166	4	31	1	166	2	31	1	158	31	2	2

23	2015	2016	2	2											600		120								4	4
24		2016	2	1	231	23	0	0	2	233	0	23	3	0	235	4	20	1	230	8	18	2				
25	2015	2016	2	1	270	57	1	0	1	271	2	59	4	1	270	3	57	0	269	4	55	1	284	57	3	3
26	2015	2016	2	1	24	13	0	0	0	24	0	13	0	0	24	1	13	0	23	0	13	0	23	13	2	2
27		2016	2	2																						
29	2015	2016	2	1																						1
30	2015	2016	3	1	205	35	0	0	0	205	0	35	0	0	205	5	35	1	202	8	34	0	197	34	4	3
31		2016	3	1																						
32	2015	2016	2	1	156	30	0	0	0	156	0	30	0	0	156	1	30	0	156	3	30	0	152	30	3	3
33	2015	2016	2	1	156	38	0	0	0	156	0	38	6	2	156	4	38	1	149	3	35	2	150	35	1	1
34	2015	2016	2	1																					3	2
35		2016	3	1																						
36	2015	2016	1	1	900	400	1	1	9	909	4	404	18	4	900	9	400	4	891	13	396	18	1129	379	4	4
37		2016	2	1																						
38	2015	2016	2	2	230	73	1	0	2	232	0	73	6	2	236	7	77	8	220	6	67	1			1	1
39	2015	2016	1	1	102	31	0	0	0	102	0	31	0	0	102	2	31	0	100	2	31	0	98	31	3	3
40		2016	2	2																						
41	2015	2016	2	1	185	20	0	0	0	185	0	20	4	0	185	0	20	0	185	3	20	1			1	1
42	2015	2016	1	1	91	7	0	0	0	91	0	7	1	0	91	0	7	1	90	3	6	0	86	5	3	3
43	2015	2016	1	2	247	51	0	0	0	247	1	52	12	0	267	11	59	9	255	9	50	3	244	41	2	2
44	2015	2016	2	1	388	74	1	0	2	390	2	76	10	1	388	7	74	2	392	6	70	2			4	3
45	2015	2016	1	1	173	31	0	0	0	173	0	31	1	0	173	3	31	1	169	1	30	0	168	30	3	3
46	2015	2016	2	1	300	35	1	0	4	304	0	35	12	1	300	6	35	2	302	5	34	0	291	35	2	2
47		2016	2	1																						
48		2016	1	1																						
49		2016	3	1	510	8	1	1	1	511	2	10	3	0	510	1	86	2	509	6	84	3				
50	2015	2016	2	2	325	30	1	0	2	327	1	31	6	2	323	8	29	5	323	6	29	3	298	36	1	1
51	2015	2016	2	1	143	44	0	0	0	143	0	44	2	0	145	2	44	2	145	1	42	0	140	41	3	3
52	2015	2016	2	1	610		1	1	3	613	0		0	0	601	3			581	25		0	575		4	4
53	2015	2016	3	1	116	17	0	0	0	116	0	17	3	0	119	2	17	1	118	2	17	2	113	19	1	1
54	2015	2016	3	1	260		0	0	1	261			16	0	276	2		0	275	11		0	317		2	
55	2015	2016	2	1	215	15	0	0	3	218	1	16	6	0	215	4	15	0	220	13	14	0	189	21	2	
56		2016	2	1																						
57	2015	2016	2	1	123	31	0	0	1	124			11	0	134	5	31	1	128	7	30	0	126	30	1	1
58	2015	2016	2	1	130	22	0	0	0	130	0	22	2	0	130	2	22	0	128	5	22	0	134	22	2	2
59		2016	2	1																						
60	2015	2016	3	1																					4	4
61	2015	2016	2	1																					3	3
62	2015	2016	1	1	447	80	1	1	4	451	0	80	6	2	455	10	82	2	447	5	80	4	424	67	4	4
63	2015	2016	2	2	83	20	0	0	0	83	0	20	4	1	87	4	21	0	86	3	21	0	77	20	1	1
64	2015	2016	1	1	135	28	0	0	2	137			1		135	1	28	0	135	1	28	0	133	29	3	3
65	2015	2016	2	1	146	21	0	0	2	148	1	22	9	0	155	2	21	0	150	3	21	0	151	21	3	3
66	2015	2016	2	2	194	32	0	0	0	194	0	32	9	1	203	7	33	1	196	5	33	2	191	30	2	2
67	2015	2016	1	1																					4	4
68	2015	2016	2	1	301	36	1	0	6	307	0	36	5	0	301	5	36	4	292	5	32	3	287	30	4	3
69	2015	2016	2	2	216	47	0	0	6	222	1	48	15	4	229	15	51	3	225	5	47	2	213	41	2	2
71	2015	2016	2	1	122	21	0	0	8	130	3	24	7	1	125	4	21	2	116	6	19	2	116	15	2	2
72		2016	3	1																						
73	2015	2016	2	1	570	242	1	1	13	583	10	252	11	2	581	12	244	9	569	9	235	41	560	194	4	4
74	2015	2016	3	1	144	20	0	0	1	145	1	21	3	0	145	2	20	0	142	1	19	0	140	20	1	1
75	2015	2016	2	2	2325	290	1	1	20	2345	3	293		5	2325	15	290	2	2305	43	287	7	2275	275	4	4
76		2016	1	1																						
77	2015	2016	1	1	171	33	0	0	0	171	0	33	5	0	175	7	33	0	168	1	33	0	167	31	1	1
78	2015	2016	2	1	342	115	1	0	4	346	5	120	7	0	349	2	115	1	347	4	114	4	335	115	4	4
80	2015	2016	3	1	160	22	0	0	1	161	0	22	3	0	165	2	22	0	164	3	22	0	156	23	2	2
81	2015	2016	2	1	92	19	0	0	0	92	0	19	2	0	92	0	19	0	92	1	19	0	88	18	3	3
82		2016	2	1																						
83	2015	2016	2	1	662	179	1	1			1	180	14	3	650	4	180	3	662	22	179	14	817	139	4	2
84	2015	2016	2	2	207	35	0	0	4	211	1	36	4	1	211	4	36	1	207	6	35	0	205	32	4	4
85	2015	2016	2	2	366	60	1	0	3	369	2	62	14	0	378	3	61	0	383	33	59	3	391		1	3
86	2015	2016	2	1	1109	293	1	1	7	1116	3	296	20	6	1102	26	290	18	1085	33	281	13	1043	259	4	4
87	2015	2016	1	1	184	37	0	0	2	186	0	37	3	1	187	4	37	0	183	8	37	3	77	11	3	3
88		2016	1	1																						
89	2015	2016	2	1	220	62	0	0	1	221	0	62	6	2	220	2	62	3	216	3	59	0	215	60	4	4
90	2015	2016	2	1	128	29	0	0	1	129	0	29	3	0	131	10	29	1	121	2	28	1	159	30	1	1
91	2015	2016	3	2	467	92	1	1	5	472	3	95	24	2	483	9	93	5	477	11	88	5	467	85	3	3
92	2015	2016	1	1	286	62	1	0	3	289	1	63	6	0	283	1	67	1	279	2	65	1	268	57	3	3
93		2016	2	1																						
94	2015	2016	1	2	242	44	0	0	3	245	2	46	5	0	245	2	45	1	242	2	43	4	213	44	2	1
95	2015	2016	3	2	120	23	0	0	1	121	0	23	3	1	120	3	30	0	116	3	30	2			4	4
96	2015	2016	2	1	120	27	0	0	0	120	0	27	3	0	123	1	27	1	122	5	26	0	114	28	3	3
97	2015	2016	1	1	370	130	1	0		370		130			370		130		370		130		395	125	4	4
98	2015	2016	1	1	400	70	1	0	3	403	1	71	5	0	400	4	70	0	397	6	69	1	398	68	2	2
99	2015	2016	1	2	95	16	0	0	0	95	0	16			95	2	17	0	93	3	17	0	96	14	3	3
100	2015	2016	1	2	153		0	0	0	153	0		6	0	153	2		0	153	1		0	157		3	
101	2015	2016	2	2	176	26	0	0	0	176	0	26	3	0	176	2	26	2	174	0	24	0	159	27	3	3
102	2015	2016	3	1	186	18	0	0	2	188	1	19	15	1	237	3	18	0	234	8	18	2	200	21	1	1
103		2016	2	2	139	34	0	0	1	140	0	34	2		139	0	34	0	138	1	34	0				
104	2015	2016	2	1	240	70	1	0	1	241	1	71	5	0	239	4	69	2	234	11	66	1			1	1
105		2016	2	1																						
106	2015	2016	2	1	63	7	0	0	2	65	0	7	0	0	55	2	7	0	53	1	7	0	57	10	4	4
107	2015	2016	2	2	31	12	0	0	0	31	0	12	1	0	30	0	12	0	31	3	12	0	27	15	1	1
108	2015	2016	2	1	267	62	1	0					10	3	279	4	65	3	276	3	62	1	272	61	1	1
110	2015	2016	2	2	116	26	0	0	2	118	0	26	6	2	115	3	30	1	113	6	30	3	104	28	1	1
111	2015	2016	3	2	116	29	0	0	4	120	1	30	12	4	112	5	28	6	107	4	21	1	99	24	4	4
112	2015	2016	1	1	352	47	1	0	1	353	0	47	0	0	325	6	47	0	346	4	47	1	338	46	4	4
113	2015	2016	3	1	101	10	0	0	1	102	0	10	0	0	101	1	10	0	101	1	10	0			2	2
114	2015	2016	1	1																					4	3
115	2015	2016	2	2	127	47	0	0	0	127	0	47	2	0	129	4	47	3	127	3	44	0	119	48	4	4
116		2016	3	2																						
117	2015	2016	2	2	254	79	1	0	4	258	1	80	25	0	274	12	78	2	261	8	75	5	225	75	1	1
118	2015	2016	2	1	98	30	0	0	0	98	0	30	0	1	128	1	30	1	98	3	30	0			4	3
119	2015	2016	2	1	411	121	1	1	2	413	0	121	1	0	423	15	121	3	411	7	116	4	393	112	4	4
120	2015	2016	2	1	186	26	0	0	2	188	0	26	8	0	190	6	26	1	185	1	25	0	184	24	1	1
121		2016	2	1	239	39	0	0	2	241			6	2	245	2	42	0	243	7	42	2	235	39		
123	2015	2016	1	1	106	44	0	0	1	107	0	44	1	0	107	1	44	3	106	0	41	0	99	41	2	2
124		2016	1	1	290	80	1	0	0	290	2	82	7	2	290	6	79	2	290	2	79	0				
125		2016	1	1	167	28	0	0	0	167	0	28	2	0	137	0	28	0	137	2	28	0	141	24		
127		2016	1	2																						
128		2016	3	1	170	30	0	0	1	171	0	30	9	0	147	7	30	0	172	1	30	0				
129	2015	2016	1	1																					4	3
130		2016	3	1	200	60	0	0	1	201			1	0	200	2	60	2	198	4	58	7				
1	2016	2017	1	1	62	51	0	0	1	63	2	53	2	0	64	3	51	1	61	2	45	1	61	45	4	4
3	2016	2017	1	1	300	60	1	0	0	306	1	69	7	0	313	8	68	2							4	3
7	2016	2017	1	1	324	90	1	0	2	325	1	90	6	0	329	4	89	2	324	5	88	4	319	84	3	2
9	2016	2017	1	2	276	38	1	0	1	276	0	38	17	1	292	13	39	0							1	1
10	2016	2017	2	1	148	69	0	0	1	150	1	70	1	0	150	3	69	3	130	0	70	2	192		4	4
11	2016	2017	2	1	255	53	1	0	1	256	0	53	11	0	266	6	53	1	248	7	53	0	237	52	3	3
12	2016	2017	1	1	175	29	0	0	2	177	1	30	1	0	176	3	29	1	143	0	38	0	142	38	4	3
13	2016	2017	3	1	730	141	1	1	6	745	4	146	6	2	745	6	144	2	740	16	125	6	714	123	4	4

14	2016	2017	3	1	156	25	0	0	3	159	0	25	0	0	156	6	25	1	130	1	24	0	129	25	3	3
15	2016	2017	3	2	196	56	0	0	1	197	2	58	13	1	209	15	57	9	182	5	37	0	177	37	1	
17	2016	2017	1	1	118	20	0	0	0	119	0	20	1	0	120	4	20	1	113	2	18	0	111	18	3	3
18	2016	2017	1	1	200	40	0	0	6	206	0	40	7	0	207	4	40	1	200	8	42	0	188	41	2	2
19	2016	2017	3	1	168	47	0	0	0	177	0	48	3	0	180	4	48	0	179	3	41	0	178	41	2	2
20	2016	2017	3	1	496	67	1	1	2	501	1	68	5	0	504	4	67	1	501	7	67	2	494	68	3	3
21	2016	2017	1	1	150	26	0	0	2	152	0	26	2	0	152	6	26	0	144	2	26	0	146	24	2	2
22	2016	2017	2	2	171	30	0	0	1	173	0	30	1	0	173	1	30	2	171	3	28	2	168	26	2	2
23	2016	2017	2	2						750		120	6	0	756	18	120	6							4	2
24	2016	2017	2	1	230	54	0	0	2	235	1	55	2	0	235	4	54	2	278	2	45	5	280	42	4	2
25	2016	2017	2	1	298	35	1	0	2	305	0	35	2	0	305	4	35	0	310	6	35	1	308	35	3	3
26	2016	2017	2	1	32	14	0	0	0	32	0	14	2	0	34	2	14	0	32	0	14	0	32	14	2	2
30	2016	2017	3	1	207	94	1	0	1	208	0	96	2	1	209	3	97	2	209	5	89	2			3	3
32	2016	2017	2	1	153	46	0	0	2	155	1	47	1	0	154	2	46	0	145	5	43	0	139	40	3	3
33	2016	2017	2	1																					1	1
36	2016	2017	1	1	219	276	1	0	1	221	4	284	3	3	223	1	283	2	350	8	370	12				
38	2016	2017	2	2	271	89	1	0	1	272	0	89	7	2	278	2	91	2	250	15	86	2	231	84	1	1
39	2016	2017	1	1	107	30	0	0	0	108	0	31	1	2	109	1	33	1	108	2	31	2	106	29	3	3
41	2016	2017	2	1	170	30	0	0	0	170	0	30	3	0	173	0	30	0							1	1
42	2016	2017	1	1	102	11	0	0	0	102	0	11	1	0	103	1	11	0	90	2	11	0	96	10	3	3
43	2016	2017	1	2	270	66	1	0	3	280	0	66	8	0	285	10	66	4	256	10	71	3	227	74	2	2
44	2016	2017	2	1	364	74	1	0	2	366	2	74	10	1	374	5	73	0	363	8	73	1	352	72	4	3
45	2016	2017	1	1	173	32	0	0	0	174	1	34	4	1	178	1	34	0	177	3	32	0	175	32	3	3
46	2016	2017	2	1	300	49	1	0	3	315	0	49	13	0	325	2	49	0	292	5	42	0	318	42	2	2
50	2016	2017	2	2	288	38	1	0	2	300	2	42	5	2	303	4	42	2	315	10	42	4	280	34	1	1
51	2016	2017	2	1	151	43	0	0	0	152	0	43	0	1	152	1	44	2	151	0	41	0	151	41	3	3
52	2016	2017	2	1	500	190	1	1		520		200	1	1	521	19	201	17							4	4
53	2016	2017	3	1	118	20	0	0	2	120	2	22	14	0	132	6	20	0	121	3	18		125	19	1	1
54	2016	2017	3	1	278	110	1	0	5	289	0	112	8	0	292	5	112	3	303	4	80	20	273	52	2	1
57	2016	2017	2	1	116	47	0	0	1	117	1	48	9	0	125	7	47	0	119	1	47	1	118	46	1	1
58	2016	2017	2	1	134	19	0	0	0	134	0	19	4	0	138	3	19	0	132	7	19	1	127	18	2	2
62	2016	2017	1	1	413	121	1	1	4	427	2	125	7	2	430	10	125	8							4	4
63	2016	2017	2	2	78	26	0	0	1	79	0	26	4	0	82	0	26	0	82	5	27	1	77	26	1	1
65	2016	2017	2	1	154	16	0	0	3	166	0	18	2	0	165	3	18	0	150	5	16	1	149	15	3	3
66	2016	2017	2	2	206	39	0	0	3	209	3	42	8	0	214	4	39	1	210	4	37	1	205	37	2	2
68	2016	2017	2	1	282	47	1	0	2	291	1	51	4	0	293	10	50	7	260	5	45	3	243	40	3	3
69	2016	2017	2	2	187	39	0	0	1	188	3	42	12	0	199	6	39	0	194	9	39	2	183	37	2	2
71	2016	2017	2	1	140	29	0	0	2	144	1	30	4	0	146	6	29	2	133	9	27	1	129	22	2	2
73	2016	2017	2	1	696	179	1	1	2	713	2	182	13	1	724	15	181	7	694	15	173	5	679	168	4	4
74	2016	2017	3	1	144	18	0	0	0	144	0	18	3	0	147	2	18	0	127	0	23	0	127	23	1	1
75	2016	2017	2	2	2130	395	1	1	60	2220	10	410	30	4	2190	15	404	5	2080	35	380	5	2050	375	4	4
77	2016	2017	1	1	154	56	0	0	2	156	3	59	4	1	158	2	57	3	161	2	53	1	158	53	1	1
78	2016	2017	2	1	434	40	1	0	7	441	1	41	7	1	441	3	41	0	473	6	30	1	437	30	4	4
80	2016	2017	3	1	160	23	0	0	0	160	0	23	6	0	166	1	23	1	160	2	22	0	158	22	2	2
81	2016	2017	2	1	95	23	0	0	1	95	0	23	4	0	98	2	23	0	96	3	21	1	95	21	3	3
83	2016	2017	2	1	957	148	1	1	4	965	2	150	22	4	983	8	152	3	711	15	141	6	704	142	4	2
84	2016	2017	2	2	190	41	0	0	1	192	0	41	4	0	195	4	41	5	190	3	36	1	187	35	4	4
85	2016	2017	2	2	422		1	0	5	427			7		429	0			334	25	120	12	0	0	3	3
86	2016	2017	2	1	1188	211	1	1	11	1192	3	212	22	5	1203	30	214	10	1152	61	190	11	1091	179	4	4
89	2016	2017	2	1	222	83	1	0	0	222	0	83	3	0	225	0	83	3	250	4	69	2	250	60	4	4
90	2016	2017	2	1	124	31	0	0	0	124	0	31	4	1	128	8	32	0	124	26	34	2	128	32	1	2
91	2016	2017	3	2	482	68	1	1	4	486	2	70	32	0	514	6	68	2	482	12	68	4	497	63	3	3
92	2016	2017	1	1	270	65	1	0	5	278	2	67	4	0	277	4	65	2	266	1	64	1	264	62	3	3
94	2016	2017	1	2	213	66	0	0	6	219	4	70	4	1	217	8	67	2	263	30			224		2	1
95	2016	2017	3	2	116	71	0	0	2	118	4	75	3	1	119	0	72	2								
96	2016	2017	2	1	134	22	0	0	1	135	0	22	2	0	136	0	22	0	133	2	22	0	131	22	3	3
97	2016	2017	1	1	390	168	1	1	0	400	0	173	0	0	400	10	173	5	350	0	170	0	345	168	4	4
98	2016	2017	1	1	420	63	1	0	3	425	2	65	6	1	428	2	64	1	415	10	64	2	404	62	2	2
99	2016	2017	1	2	105	13	0	0	0	105	0	13	0	0	105	2	13	0	89	5	15	0	90	14	4	4
100	2016	2017	1	2	135	12	0	0	0	153	0	12	5	0	158	1	12	0	152	4	11	0	148	11	3	3
101	2016	2017	2	2	199	20	0	0	3	202		20	1	1	200	4	21	1	175	10	18	0	164	19	1	1
102	2016	2017	3	1	165	46	0	0	2	187	1	50	15	2	200	3	51	0	172	5	65	0	162	65	1	1
103	2016	2017	2	2																						
104	2016	2017	2	1	281		0	0	8	289			3		284	14				16		20	188	58	1	1
106	2016	2017	2	1	60	5	0	0	0	64	0	5	1	0	65	1	5	0	58	4	5	0	49	9	3	3
107	2016	2017	2	2	34	9	0	0	0	38	0	9	2	0	40	1	9	0	45	2			43	0	1	1
108	2016	2017	2	1																					1	1
110	2016	2017	2	2	115	23	0	0	2	115	0	23	3	1	116	0	24	0	143	5			120	23	1	1
111	2016	2017	3	2	90	7	0	0	3	93	0	7	6	0	96	8	7	1	90	1	6	0	85	6	4	4
112	2016	2017	1	1	324		1	0	9	333	0		2	0	326	2		0	331	1			329	0	4	
113	2016	2017	3	1	92	12	0	0	3	97	0	14	2	1	96	0	15	0	97	0	12	0	94	12	3	3
115	2016	2017	2	2	135	50	0	0	1	136	1	51			135	13	50	9	120	0	43	0			4	4
117	2016	2017	2	2	255	73	1	0	5	251	0	73	16	1	262	10	74	6	259	2	67	0	256	67	1	1
118	2016	2017	2	1	167	53	0	0		178	2	56	1	1	179	4	55	3	166	11	48	2	109	46	4	3
119	2016	2017	2	1	430	97	1	1	2	438	0	110	5	0	441	8	110	1	427	7	96	1	425	94	4	4
120	2016	2017	2	1	193	20	0	0	2	193	0	21	10	0	201	5	21	0	200	10	20	0	187	20	1	1
121	2016	2017	2	1	258	41	0	0	1	259	1	42	10	2	268	1	43	2	264	6	39	2	254	39	2	2
123	2016	2017	1	1	122	37	0	0	0	122	0	37	3	1	125	4	38	2	118	6	34	0	112	34	2	2
124	2016	2017	1	1	320	75	1	0	1	330	0	77	1	0	330	9	77	0	350	10	95	5	350	90	3	3
125	2016	2017	1	1	167	26	0	0	0	170	0	26	2	0	172	1	26	1	233	2	25	0	233	25	4	4
128	2016	2017	3	1	153	32	0	0	1	153	0	33	9	0	161	5	33	0	161	6	29	1	155	28	1	1
129	2016	2017	1	1	265	48	1	0	1	266	1	49	3	0	268	1	48	1	267	5	47	0	262	47	4	3
